# Supplementary material for: Cooperation and partner choice among Agta hunter-gatherer children: An evolutionary developmental perspective
Source: PLoS One. 2023 Apr 26;18(4):e0284360. doi: 10.1371/journal.pone.0284360 (PMC10132543; doi:10.1371/journal.pone.0284360)
Supplement: S1 File — This supplementary information file contains additional information regarding the aging method (section S1), additional details of the children’s cooperative game methods (section S2), a summary of the methods for the cooperative games played with Agta adults (section S3), plus all supplementary tables (S1-S9 Tables) and figures (S1-S16 Figs). (PDF) [file pone.0284360.s001.pdf]

Supplementary material for ‘Cooperation and partner choice among Agta hunter-gatherer children: an evolutionary developmental perspective’

Daniel Major-Smith, Nikhil Chaudhary, Mark Dyble, Katie Major-Smith, Abigail E Page, Gul Deniz Salali, Ruth Mace & Andrea B Migliano

Section S1: Description of the aging method

As ages for most Agta were unknown, a method to estimate ages was developed, which improves upon previous methods of age estimation. A Bayesian method was developed, which takes two sources of information as input: relative age rankings and *a priori* age distributions for each individual. We briefly describe the method here, with further detail provided in the following paper [1].

During fieldwork, we constructed relative age rankings by first printing photographs of all individuals, which were then grouped into approximate age cohorts (0-4, 4-8, 8-12, 39-19, 20-45 and 45+ years; with those not easily assigned to one cohort included in the two nearest groups). Either individually or in small groups, Agta individuals were then presented with photographs of individuals from a target cohort one at a time. The target cohort was the age group the interviewee was in, in addition to all cohorts younger than themselves. Individuals from a specific camp were presented with photos of Agta from both their camp and neighbouring camps. More distant camps were not included due to a lack of familiarity, unless the interviewee knew individuals from more distant camps well (e.g., they grew up in the distant camp). The interviewee’s photo was displayed first (unless they were not in the target cohort, in which case another photo was selected), after which subsequent photos were presented one at a time. Participants were first asked if they knew the target individual. If so, they were then asked if they were familiar enough with the target to know their approximate age relative to other individuals. Each photograph was put into one of three categories: ‘don’t know’, ‘know but not the age’ and ‘know with age’. If the interviewee knew both the target and their age, they were asked to rank the age of the target individual relative to others (with left meaning younger and right meaning older). This method produced a relative age list from youngest to oldest. This process was repeated multiple times, producing a total of 266 partial ranks which encompassed 587 Agta.

The second stage of the ageing process involved deriving age estimates for each of these individuals. This was based on various sources of information – with varying degrees of accuracy – such as external databases with Agta names and age/date of birth information [2], birth records, self-reported age/date of birth, triangulation to known events (e.g., martial law in 1970, various known typhoons, age-mates with known birthdates), school grade and dental development (for children), and – if there was little information to go on – the best guesses of the researchers. Of these 587 Agta, 98 were given an exact birthday, while 93 had an age estimate +/- 1 year. For the rest of the Agta without known ages, four researchers who worked with the Agta

estimated upper and lower age ranges; the highest and lowest age ranges were then used going forward.

These two sources of information – the multiple relative age lists and age estimates – were then combined together using a Bayesian Gibbs sampling approach. This approach took these relative age lists and combined them together if possible, and then randomly-selected an age value for each individual from their *a priori* age distribution consistent with the relative ranking. This process was then iterated 15,000 times on each relative age list, producing the final age distributions for each individual. Although each individual had an associated age distribution, for simplicity this was reduced to a point estimate for analyses here, using the mean value. Using a validated dataset of known ages, this approach was found to assign more accurate ages than previously-used methods for age estimation.

Children without ages assigned via this procedure (~25% of children) were given an age estimate based on birth records (if available), reference to children with known ages, and/or the best guess of the researcher. As ages of children are easier to estimate with greater certainty than for adults – combined with the fact that many of the children without ages assigned via the Bayesian approach were from camps with greater integration with the local economy, who were more aware of their child's age – any measurement error is likely to be relatively small (e.g., a 12 year-old without a known age or date of birth could perhaps be mistaken for a 10 or 14 year-old, but not a 8 or 16 year-old).

Section S2: Additional details on study methods

Initial piloting of this methodology was tested on a small number of children in the first field season (between April and June 2013). The pilot study seemed successful, with all children appearing to understand the game and no confusion over the rules. The same methodology was then employed in the second field season (between February and October 2014) to as many Agta children as possible in all the camps visited.

Field assistants were briefed on the aims of the study and trained in the methodology. As the games were intentionally very simple, and the field assistants had experience conducting similar games with adults, they quickly learned how to conduct the children's games. For all games, the lead author (DM-S) was present and explained the rules of the game to the participant, which were then translated by the field assistant. As such, with the experimenter present it was possible to see the reactions of the children and whether they appeared to understand the game. The majority of the children's games were conducted with the help of one of two field assistants, in addition to the experimenter.

The following script is representative of how the children's games were conducted (with actions to be performed by the experimenter indicated in square brackets):

"We are going to play a simple game where you can win candy both for yourself and for your camp-mates. Here are five candies [show candies]. For each candy, you have to decide first if you would like to keep it for yourself, or if you would like to give it to a camp-mate [with the candy, motion towards the participant and then towards the wider camp]. Secondly, if you decide to give it to a camp-mate, which camp-mate? Any candies you keep for yourself, you will get to keep. Any candies you give to others, they will get to keep. We will give these candies out after we have played the games with everyone in camp. There are no correct answers in this game, so you can give as many or as few candies as you want, to whichever camp-mates you want. No-one else in camp will know how many you kept or who you gave to.

Do you understand? Do you have any questions?

For the first candy [show candy], would you like to keep it for yourself, or give it to a camp-mate? [If chooses a camp-mate, ask:] Which camp-mate would you like to give this candy to? [Write decision in notebook and set the first candy to one side].

For the next candy [show candy], would you like to keep it for yourself, or give it to a camp-mate? [If chooses a camp-mate, ask:] Which camp-mate would you like to give this candy to? [Write decision in notebook and set the second candy to one side].

[Repeat until only one candy left]

For the last candy [show candy], would you like to keep it for yourself, or give it to a camp-mate? [If chooses a camp-mate, ask:] Which camp-mate would you like to give this candy to? [Write decision in notebook and set the final candy to one side].

Okay, that's it! The candies will be given out soon once we have played games with all children in camp. It is also very important that you do not tell anyone else how you played in this game – *Secreto*! [The Tagalog word for 'secret'].

Thank you again for taking part!"

Section S3: Details of the cooperative game played with adults

As noted in the main text, similar cooperative games were also played with adult Agta, from which the adult levels of cooperation were taken. This method has been described in detail previously [3], but will be summarised again here.

For this adult game, participants were shown photos of themselves, along with a maximum of 10 randomly-selected camp-mates (for camps with 10 or fewer other camp-mates, participants were shown all other camp-mates). Participants were then given a number of small wooden tokens, each representing a portion of rice (approx. 125 grams), equal to the number of camp-mate's photos. This number of tokens was chosen to cause a social dilemma as to whether to share and with whom, as it was not possible for participants to give resources to all camp-mates *and* themselves. Participants were then given each token one at a time, and asked whether they wanted to keep the rice for themselves (by putting the token on their own photo), or to give the rice to a camp-mate, and, if so, who this person was (by putting the token on their photo). This was repeated until there were no tokens remaining. The measure of cooperation was therefore the percentage of resources that were shared with others, ranging from 0% (no resources shared) to 100% (all resources shared).

Note that in addition to the game described above – known as the 'Sharing Game' – adults also played another game – known as the 'Taking Game' – which involved taking resources *from* the photos of their camp-mates. Although cooperative behaviour in both games was highly correlated [3], because of its similarity to the game played with children we decided to use the results of the 'Sharing Game' as the measure of adult cooperation to compare against children's cooperative behaviour.

References cited in supplementary information

1. Diekmann Y, Smith D, Gerbault P, Dyble M, Page AE, Chaudhary N, et al. Accurate age estimation in small-scale societies. Proc Natl Acad Sci U S A. 2017;114: 8205–8210. doi:10.1073/pnas.1619583114

2. Headland TN, Headland JD, Uehara RT. Agta Demographic Database: Chronicle of a hunter-gatherer community in transition. SIL Language and Culture Documentation and Description, 2.; 2011.

3. Smith D, Dyble M, Thompson J, Major K, Page AE, Chaudhary N, et al. Camp stability predicts patterns of hunter-gatherer cooperation. R Soc Open Sci. 2016;3: 160131. doi:https://doi.org/10.1098/rsos.160131

Table S1: Descriptive statistics of participants per camp (*n*=179; camps=14). The final row gives the summary statistics over the entire sample. SD = standard deviation; SE = standard error.

**Camp code**	***n***	***n* males (%)**	**Mean age (SD)**	**Age range**	**Mean relatedness (SD)**	**Relatedness range**	**Average adult level of cooperation in camp (SE)**
P1	3	1 (33.3%)	8.7 (1.4)	7.2 to 10.1	0.190 (0.099)	0.075 to 0.247	59.2% (3.73)
P2	46	24 (52.2%)	9.9 (4.1)	3.0 to 18.0	0.053 (0.023)	0.011 to 0.093	31.4% (3.61)
P3	14	9 (64.3%)	9.4 (2.7)	4.8 to 13.3	0.096 (0.044)	0.012 to 0.131	52.8% (3.47)
P4	7	4 (57.1%)	10.7 (1.9)	8.1 to 14.3	0.075 (0.038)	0.014 to 0.128	56.9% (7.34)
P5	16	9 (56.2%)	8.1 (2.4)	4.5 to 12.6	0.090 (0.029)	0.053 to 0.121	38.5% (6.44)
P6	17	7 (41.2%)	7.5 (2.7)	3.5 to 13.6	0.119 (0.039)	0.000 to 0.154	21.7% (5.19)
P7	22	8 (36.4%)	9.5 (2.5)	5.4 to 15.1	0.076 (0.029)	0.029 to 0.116	28.2% (4.28)
P8	6	2 (33.3%)	5.8 (1.7)	4.3 to 7.9	0.156 (0.077)	0.050 to 0.223	6.7% (6.67)
P9	9	5 (55.6%)	8.2 (2.1)	6.0 to 12.0	0.113 (0.036)	0.069 to 0.153	0.0% (0.00)
P10	17	10 (58.8%)	9.0 (3.3)	4.2 to 13.9	0.091 (0.029)	0.041 to 0.122	30.4% (6.61)
P11	6	2 (33.3%)	8.9 (2.6)	6.3 to 12.9	0.127 (0.038)	0.062 to 0.160	59.0% (5.47)
M1	6	4 (66.7%)	7.2 (1.3)	6.1 to 9.1	0.099 (0.046)	0.040 to 0.152	69.3% (4.52)
M2	7	1 (14.3%)	6.3 (2.1)	4.0 to 10.0	0.140 (0.047)	0.033 to 0.158	49.3% (7.81)
M3	3	1 (33.3%)	11.7 (1.5)	10.0 to 13.0	0.223 (0.008)	0.214 to 0.228	60.7% (9.64)
**Total**	**179**	**87 (48.6%)**	**8.9 (3.2)**	**3.0 to 18.0**	0.092 (0.050)	0.000 to 0.247	40.3% (5.69)

Table S2: Results of multi-level Poisson and ordinal regression models regarding the factors associated with the number of candies given by children (*n*=179, camps=14). Both the univariable (separate) and multivariable (combined) model results are presented here. Positive parameter estimates indicate an increase in the number of resources given to others. Parameter estimates of Poisson regression models are given in incidence rate ratios and are interpreted as the increased rate ratio of children sharing an extra gift given a one-unit increase in the predictor variable (e.g., based on the multivariable model, a one-unit increase in adult camp average increases the rate ratio of children sharing an additional resource by 1.022, meaning that the rate of children sharing increases by 2% for each unit increase in adult cooperation). Parameter estimates of the ordinal regression models are given in odds ratios and are interpreted as the likelihood of an increase in the amount given by children for every one-unit increase in the predictor variable (e.g., based on the multivariable model, a one-unit increase in adult camp average increases the odds of sharing more resources by 1.050, meaning that the odds of children sharing increases by 5% for each unit increase in adult cooperation). Due to the small number of children sharing 5 resources (Table 1), for the ordinal regression models the categories '4' and '5' were combined together. Note also that the credible intervals for the 'relatedness' term in both the Poisson and ordinal regression models are very wide, meaning it is poorly-estimated and therefore likely to be highly inaccurate.

**Variable**	**Model**	**Level**	***Poisson models***		***Ordinal regression models***	
			**Incidence rate ratio**	**95% credible intervals**	**Odds ratio**	**95% credible intervals**
Age	Univariable	Individual	1.032	0.995; 1.070	1.098	1.001; 1.207
Sex (1 = male)	Univariable	Individual	1.185	0.953; 1.483	1.572	0.896; 2.765
Relatedness	Univariable	Individual	0.446	0.024; 8.171	0.117	0.000; 137.306
Adult coop	Univariable	Camp	1.023	1.008; 1.039	1.052	1.021; 1.088
Age	Multivariable	Individual	1.029	0.992; 1.067	1.096	0.998; 1.208
Sex (1 = male)	Multivariable	Individual	1.174	0.935; 1.473	1.616	0.902; 2.874
Relatedness	Multivariable	Individual	0.421	0.028; 6.270	0.103	0.000; 94.651
Adult coop	Multivariable	Camp	1.022	1.008; 1.041	1.053	1.018; 1.096

Table S3: Results of multi-level Poisson and ordinal regression models regarding whether maternal (*n*=155), paternal (*n*=145) and joint-parental (*n*=162) levels of cooperation were associated with the number of candies given by children (camps=14). For the joint-parental cooperation model, the average of mother's and father's cooperation was used (if only one parent participated, just their data was used). All models are univariable analyses. Positive parameter estimates indicate an increase in the number of resources given to others (see the legend of Table S2 for a more detailed explanation of interpreting the parameter estimates from the Poisson and ordinal regression models). Due to the small number of children sharing with 5 unique recipients (Table 1), for the ordinal regression models the categories '4' and '5' were combined together.

**Variable**	***Poisson models***		***Ordinal regression models***	
	**Incidence rate ratio**	**95% credible intervals**	**Odds ratio**	**95% credible intervals**
Mother cooperation	1.002	0.996; 1.009	1.008	0.993; 1.023
Father cooperation	0.997	0.991; 1.002	0.991	0.976; 1.005
Joint-parental cooperation	0.999	0.993; 1.006	0.997	0.981; 1.014

Table S4: Results of multi-level linear, Poisson and ordinal regression models regarding the factors associated with the number of unique recipients children shared with (*n*=179, camps=14). Both the univariable (separate) and multivariable (combined) model results are presented here. Positive parameter estimates indicate an increase in the number of unique individuals the participant shared with (see the legend of Table S2 for a more detailed explanation of interpreting the parameter estimates from the Poisson and ordinal regression models). Due to the small number of children sharing with 5 unique recipients (Table 1), for the ordinal regression models the categories '4' and '5' were combined together. Note also that the credible intervals for the 'relatedness' term in both the Poisson and ordinal regression models are very wide, meaning it is poorly-estimated and therefore likely to be highly inaccurate. Incorporating variation in average adult camp levels of cooperation into account did not alter associations in either the univariable (*b_adult* = 0.034, 95% credible interval = [0.018, 0.050]) or multivariable (*b_adult* = 0.033, 95% credible interval = [0.014, 0.051]) linear regression models.

Variable	Model	Level	***Linear models***		***Poisson models***		***Ordinal regression models***	
			**Parameter estimate (SE)**	**95% credible intervals**	**Incidence rate ratio**	**95% credible intervals**	**Odds ratio**	**95% credible intervals**
Age	Univariable	Individual	0.101 (0.030)	0.043; 0.159	1.067	1.025; 1.109	1.173	1.066; 1.293
Sex (1 = male)	Univariable	Individual	0.131 (0.182)	-0.226; 0.492	1.089	0.851; 1.383	1.311	0.757; 2.289
Relatedness	Univariable	Individual	-2.416 (2.408)	-7.132; 2.264	0.179	0.009; 4.087	0.009	0.000; 9.736
Adult coop	Univariable	Camp	0.034 (0.008)	0.018; 0.051	1.026	1.013; 1.041	1.059	1.031; 1.094
Age	Multivariable	Individual	0.093 (0.029)	0.036; 0.151	1.062	1.019; 1.105	1.168	1.062; 1.287
Sex (1 = male)	Multivariable	Individual	0.125 (0.178)	-0.227; 0.476	1.082	0.857; 1.381	1.423	0.792; 2.564
Relatedness	Multivariable	Individual	-1.811 (2.243)	-6.256; 2.435	0.224	0.012; 3.730	0.021	0.000; 24.776
Adult coop	Multivariable	Camp	0.033 (0.009)	0.014; 0.051	1.026	1.012; 1.044	1.062	1.025; 1.107

Table S5: Additional robustness checks on the model with relatedness as the response variable and age and sex as predictors, given that the assumptions of normality and equal variances are likely to be violated (Figs S11 and S12). Initially, we converted 'relatedness' into a binary variable of 'shared with siblings (r=0.5)' (n=160) vs 'shared with other camp-mates (r<0.5)' (n=148) and performed a logistic mixed-effects model with age and sex as predictor variables and participant ID as a random effect. However, the estimates from these models were highly implausible and contained large error terms. For instance, in this model boys had an estimated 21 times greater odds of giving to siblings compared to girls, with exceptionally wide credible intervals (95% CI = [2.23; 382.70]), while for age there was an estimated 40% reduction in the odds of sharing with siblings per year of age (95% CI = [0.37; 0.84]). The predicted values from this model also appeared unrealistic, given the raw data; for example, although young boys were more likely to share with siblings, the model predicted that girls aged 5, 10 and 15 had 72%, 21% and 3% predicted probabilities of sharing with only siblings, respectively, while for boys aged 5, 10 and 15 the equivalent probabilities were 97%, 79% and 32%. These estimates and predicted values are highly improbable, suggesting estimation and convergence issues with the model.

In an attempt to provide more reasonable estimates, rather than using default non-informative priors, we applied informative priors to the fixed-effect estimates for the intercept, participant age, and participant sex terms. These priors were taken from a single-level model which, while not taking the random effects structure into consideration, provided plausible estimates to work from. For the intercept prior, we set this as a normal distribution with a mean log-odds of 1 (equivalent to an odds ratio of 2.7), with a standard deviation of 0.5. For the participant age prior, we set this as a normal distribution with a mean log-odds of -0.1 (equivalent to an odds ratio of 0.9), with a standard deviation of 0.2. For the participant sex prior, we set this as a normal distribution with a mean log-odds of 0.8 (equivalent to an odds ratio of 2.2), with a standard deviation of 0.5. The results of this model were more plausible, with increasing age predicting a decrease in the probability of sharing with siblings (odds ratio = 0.83, 95% CI = [0.73; 0.95]), and boys being more likely to share with siblings than girls (odds ratio = 3.58, 95% CI = [1.47; 8.49]). The predicted values from this model were also more sensible and congruent with the raw data, compared to the model with non-informative priors, with the model predicting that girls aged 5, 10 and 15 had 58%, 35% and 20% predicted probabilities of sharing with only siblings, respectively, while for boys aged 5, 10 and 15 the equivalent probabilities were 83%, 64% and 44%.

To further test the robustness of these results, single-level models were performed based on the average relatedness to recipients (rather than each recipient as a separate data point). This entailed a loss of data, but greatly simplified the modelling procedure as each individual only possessed one observation, removing the need for mixed-effects models. This simplified dataset contained 125 observations. The results of this linear model are presented in the table below under the 'linear model' column. These results are qualitatively identical to those of the linear mixed-effects model reported in the main text. However, even though the assumption of equal variances appears met in this model, the assumption of normality still appears to be violated (Fig S13).

A further model was therefore constructed by dichotomising average relatedness into ‘only gave to siblings (average *r* = 0.5)’ and ‘did not only share with siblings (average *r* < 0.5)’. 56 children only gave to siblings, while 69 children did not exclusively share with siblings. These results are presented in the ‘logistic model’ column of the table below and again report a strong effect of age, with older children less likely to only share with siblings. A strong effect of sex was reported here too, with boys having approximately 3 times greater odds of sharing only with siblings. To help interpret these odds ratios, the following predicted probabilities of sharing with only siblings were estimated based on this model: girls aged 5, 10 and 15 had 53%, 26% and 12% predicted probabilities of sharing with only siblings, respectively, while for boys aged 5, 10 and 15 the equivalent probabilities were 77%, 53% and 27%.

Although the route was rather convoluted, these findings do suggest that the results in the main text of recipient relatedness decreasing with age is robust, and that boys were somewhat more likely to share with closer kin.

	**Linear model (*n*=125)**		**Logistic model (*n*=125)**	
**Variable**	**Parameter estimate**	**95% credible interval**	**Odds ratio**	**95% credible interval**
Intercept	0.416	0.308; 0.522	3.474	0.944; 13.094
Participant age	-0.015	-0.026; -0.005	0.795	0.692; 0.910
Participant sex (1 = male)	0.062	-0.004; 0.129	3.319	1.515; 7.499

Table S6: Supplementary analyses exploring associations between participant age, participant sex and relatedness (and their interactions) with 'recipient age' as the response variable (*n*=308 gifts from 125 children). The average recipient age was 6.8 years (SD = 3.9), with a range from 0.3 to 18.0 years. Relatedness was strongly associated with the age of recipient, with lower levels of relatedness associated with sharing with older recipients (that is, donations to younger children were towards siblings and other close kin, while donations to older children were towards distant kin or non-relatives). A positive relationship between participant and recipient age was also found, with older children more likely to share with other older children; a 10-year increase in participant age predicted approximately a 2-year increase in age of the recipient. Plots of these associations, based on predicted recipient age values, are given in Figs S14 and S15 for participant age and relatedness, respectively. No association with participant sex was reported. No strong interactions were identified (age by sex = 0.27, 95% CI = [-0.07; 0.60]; age by relatedness = -0.03, 95% CI = [-0.71; 0.63]; sex by relatedness = 3.84, 95% CI = [-0.36; 8.13]). Tests of normality and homoskedasticity based on the main effects model (with no interactions) can be found in Fig S16 and suggests that both of these assumptions are likely to be satisfied.

**Variable**	**Parameter estimate**	**Standard error**	**95% credible intervals**
Intercept	5.862	1.008	3.855; 7.857
Age	0.222	0.088	0.052; 0.393
Sex (1 = male)	0.126	0.543	-0.957; 1.191
Relatedness	-4.087	1.152	-6.288; -1.838

Table S7: Supplementary analyses exploring associations between participant age, participant sex and relatedness (and their interactions) with 'age difference between participant and recipient' as the response variable (*n*=308 gifts from 125 children). The average age difference was -2.3 years (SD = 4.4), indicating that most recipients were younger than the participant, with a range from -17.3 to 10.5 years. A strong effect of participant age was found, with the age gap between participant and recipient increasing with participant age. This may seem contrary to the previous result that recipient age increased with participant's age (Table S6), but both statements are true. For instance, 6-year olds were predicted on average to give to other 6-year olds, while 14-year olds were predicted on average to give to 8-year olds (Fig S14), meaning that both recipient age (from 6 to 8) and the age gap (from 0 to -6) increased with participant's age.

An increase in relatedness was also associated with an increased age difference, indicating that participants were more likely to give to children younger than themselves if the recipient was closer kin, while less closely-related recipients were of a more similar age. No association with participant sex was reported. Note that the 'relatedness' and 'sex' coefficients from these 'recipient age' and 'age difference' models are practically identical; this is because the recipient age model (Table S6) and the age difference model (here) are essentially measuring the same thing, given that recipient age is simply the age difference plus participant's age.

No strong interactions were identified (age by sex = 0.27, 95% CI = [-0.07; 0.61]; age by relatedness = -0.04, 95% CI = [-0.70; 0.60]; sex by relatedness = 3.91, 95% CI = [-0.30; 8.33]). Tests of normality and homoskedasticity based on the main effects model (with no interactions) can be found in Fig S16 and suggests that both of these assumptions are likely to be satisfied.

**Variable**	**Parameter estimate**	**Standard error**	**95% credible intervals**
Intercept	5.852	1.019	3.838; 7.818
Age	-0.776	0.089	-0.946; -0.600
Sex (1 = male)	0.125	0.549	-0.959; 1.190
Relatedness	-4.060	1.110	-6.212; -1.845

Table S8: Supplementary analyses exploring associations between participant age, participant sex and relatedness (and their interactions) with 'recipient sex' (1 = male) as the response variable (*n*=308 gifts from 125 children). Of 308 nominations, 164 (53.2%) were to males and 144 (46.8%) to females. A strong effect of participant sex was found, with children having approximately five times the odds of choosing recipients of the same sex. To help interpret these odds ratios, using average participant age and relatedness values, the predicted probability of a girl sharing with a boy was 38%, while for boys the predicted probability of a boy sharing with another boy was 73%. No strong effects of participant age or relatedness were reported.

No interactions between age and sex (odds ratio = 0.90, 95% CI = [0.65; 1.23]) or sex and relatedness (odds ratio = 0.19, 95% CI = [0.003; 9.63]) were found for this outcome. However, an interaction between age and relatedness was found (odds ratio = 0.45, 95% CI: [0.21; 0.85]). Older individuals were more likely to share with boys when relatedness was low, while they were less likely to share with boys if relatedness was higher. For instance, the predicted probabilities of a girl nominating a boy when relatedness was 0 were 24%, 48% and 73% at ages 5, 10 and 15, respectively; while if relatedness was 0.5, then the predicted probabilities were 44%, 27% and 16%. The predicted probabilities for boys tells a similar story. At ages 5, 10 and 15, the probabilities of nominating another boy when relatedness was 0 were 58%, 82% and 92%; while if relatedness was 0.5 then the predicted probabilities were 78%, 64% and 46%.

**Variable**	**Odds ratio**	**95% credible intervals**
Intercept	0.955	0.178; 5.424
Age	1.000	0.857; 1.157
Sex (1 = male)	4.821	1.808; 14.414
Relatedness	0.208	0.028; 1.355

Table S9: Supplementary analyses exploring associations between participant age, participant sex and relatedness (and their interactions) with ‘sex similarity’ (1 = same sex) as the response variable (*n*=308 gifts from 125 children). Of 308 nominations, 187 (60.7%) were to same-sex recipients and 121 (39.2%) opposite-sex recipients. No effects of participant age, participant sex or relatedness were reported. No strong interactions were identified either (age by sex odds ratio = 1.02, 95% CI = [0.74; 1.37]; age by relatedness odds ratio = 0.86, 95% CI = [0.47; 1.57]; sex by relatedness odds ratio = 0.03, 95% CI = [0.0004; 1.59]).

**Variable**	**Odds ratio**	**95% credible intervals**
Intercept	3.177	0.597; 18.498
Age	0.946	0.814; 1.094
Sex (1 = male)	1.736	0.699; 4.637
Relatedness	0.497	0.070; 3.288

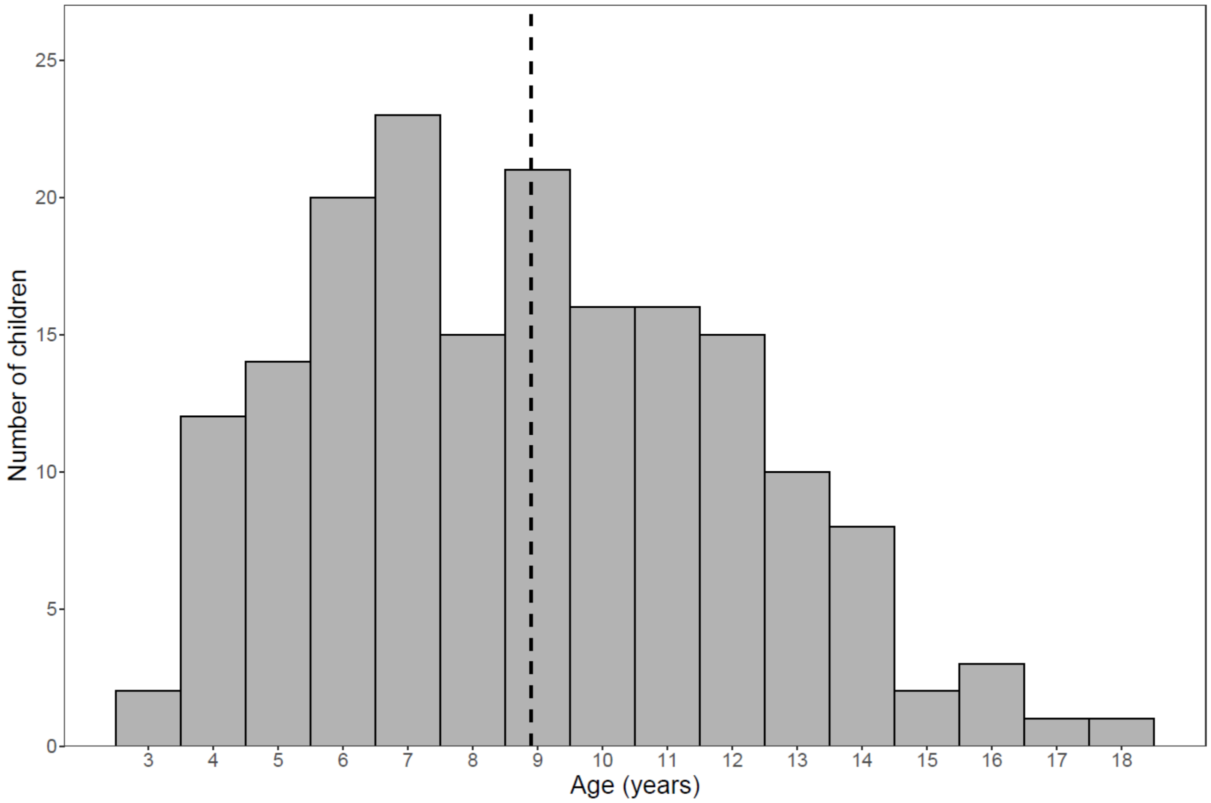

Fig S1: Histogram of child ages (*n*=179). The dashed line indicates the mean age value.

Fig S2: Directed Acyclic Graph (DAG) representing assumed causal relations between variables in the 'amount shared' analyses, with child levels of cooperation ('childCoop') as the outcome.

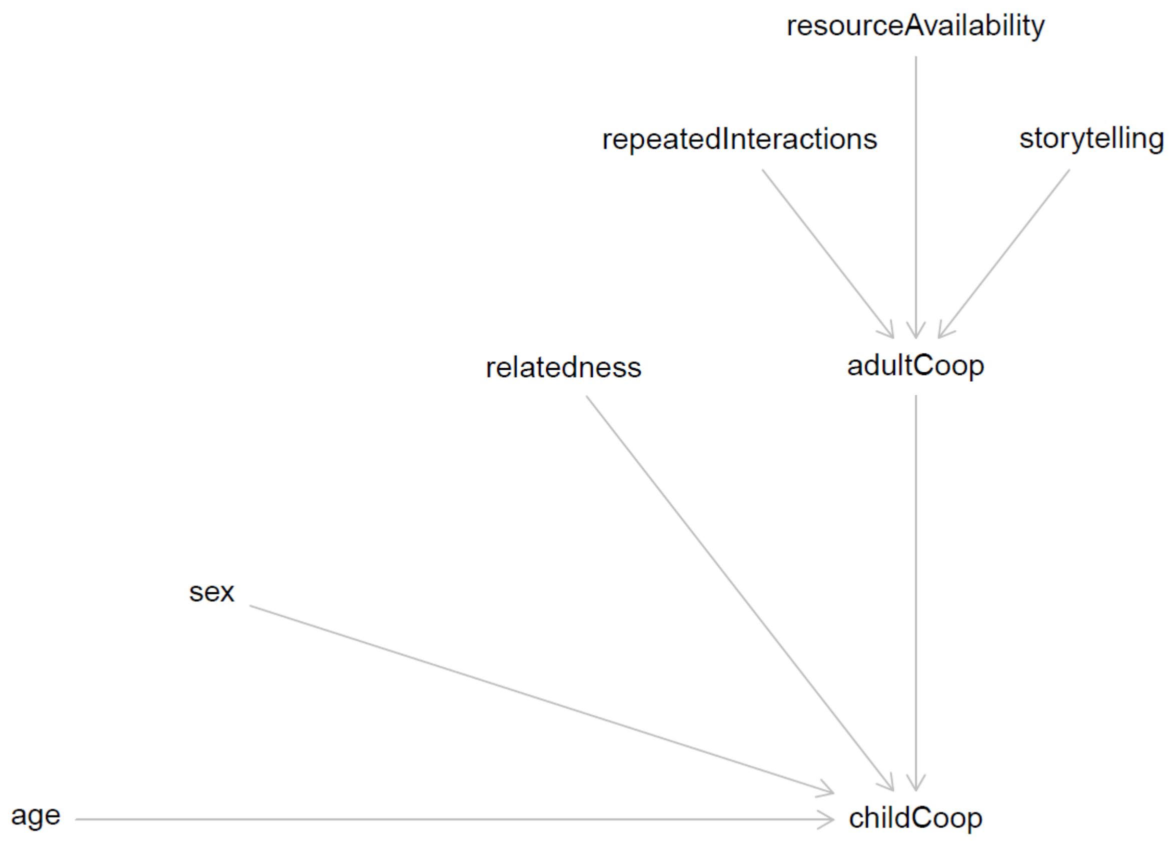

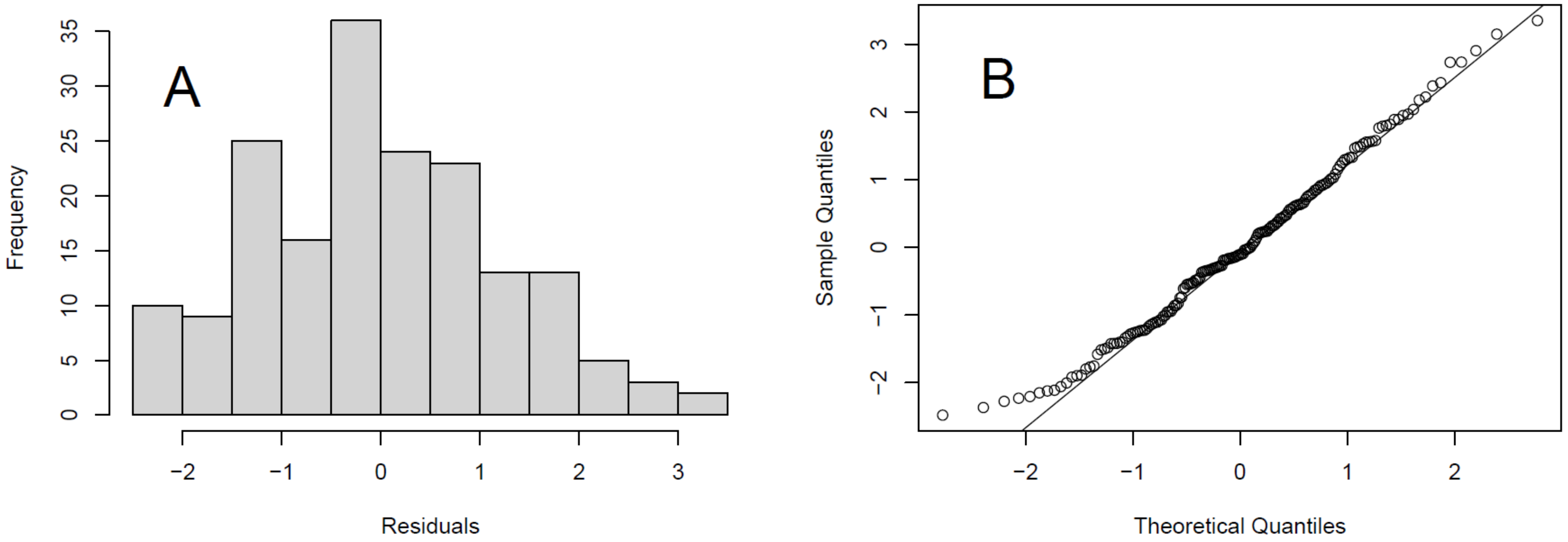

Fig S3: Testing the assumption of normality of the linear multivariable multi-level model with 'total number of resources shared' as the response variable, age, sex, average relatedness to child camp-mates, and average adult level of cooperation in camp as fixed effects and camp as a random effect. Both the histogram (A) and the Q-Q plot (B) indicate that the residuals of this models are approximately normally distributed, meeting the assumption of normality. Sensitivity analyses using multi-level Poisson and ordinal regression methods (which do not rely on assumptions of normality) find equivalent patterns of results to those of the linear multi-level model in Table 2, suggesting that the conclusions reported in the main text are robust (Table S2).

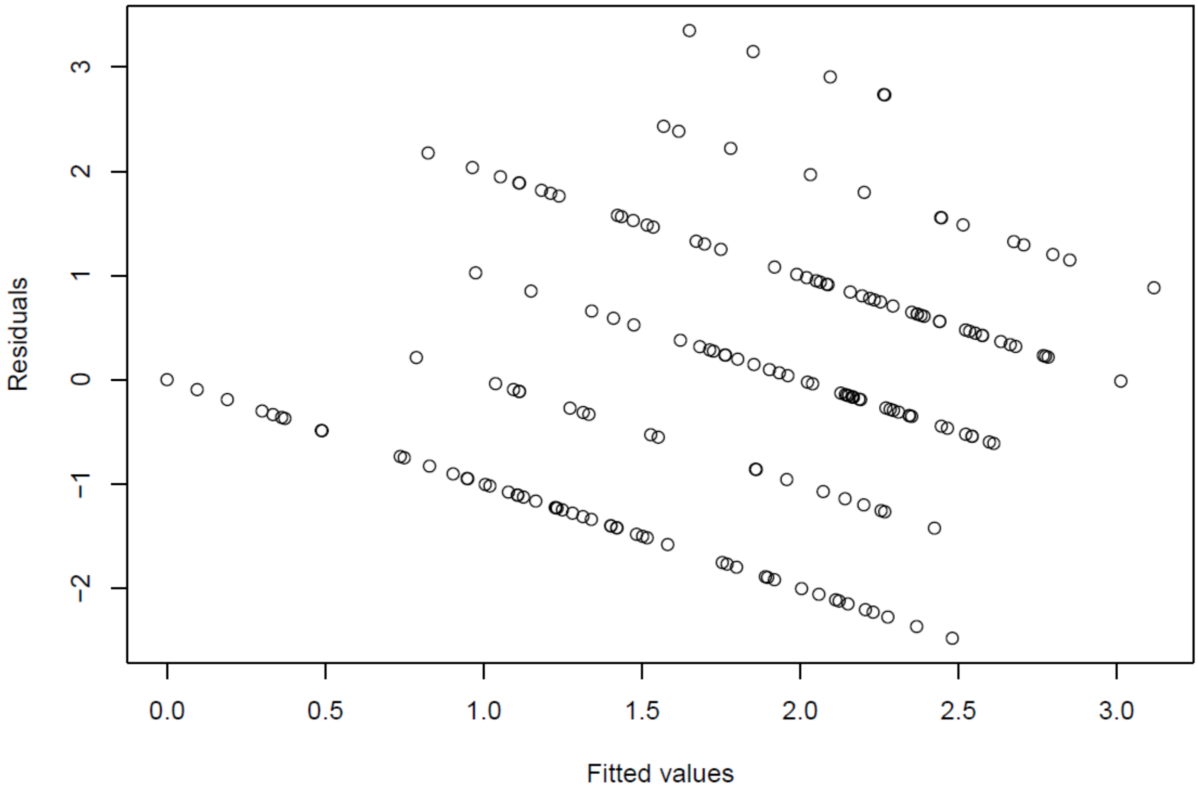

Fig S4: Testing the assumption of homoskedasticity of the linear multivariable multi-level model with ‘total number of resources shared’ as the response variable, age, sex, average relatedness to child camp-mates, and average adult level of cooperation in camp as fixed effects and camp as a random effect. This plot compares the residuals of this multivariable model against the fitted values. No strong ‘fan shape’ effects are apparent, meaning that the variance in residuals is approximately uniform for all fitted values (other than for extreme fitted values; below 0.5 and above 2.5, where the residual variance does appear to be lower). Nonetheless, given that the raw data for ‘total number of resources shared’ could only take on six possible values (between 0 and 5), the interpretation of this plot regarding the assumption of homoskedasticity is equivocal. However, sensitivity analyses using multi-level Poisson and ordinal regression methods (which do not rely on assumptions of homoskedasticity) find equivalent patterns of results to those of the linear multi-level model in Table 2, suggesting that the conclusions reported in the main text are robust (Table S2).

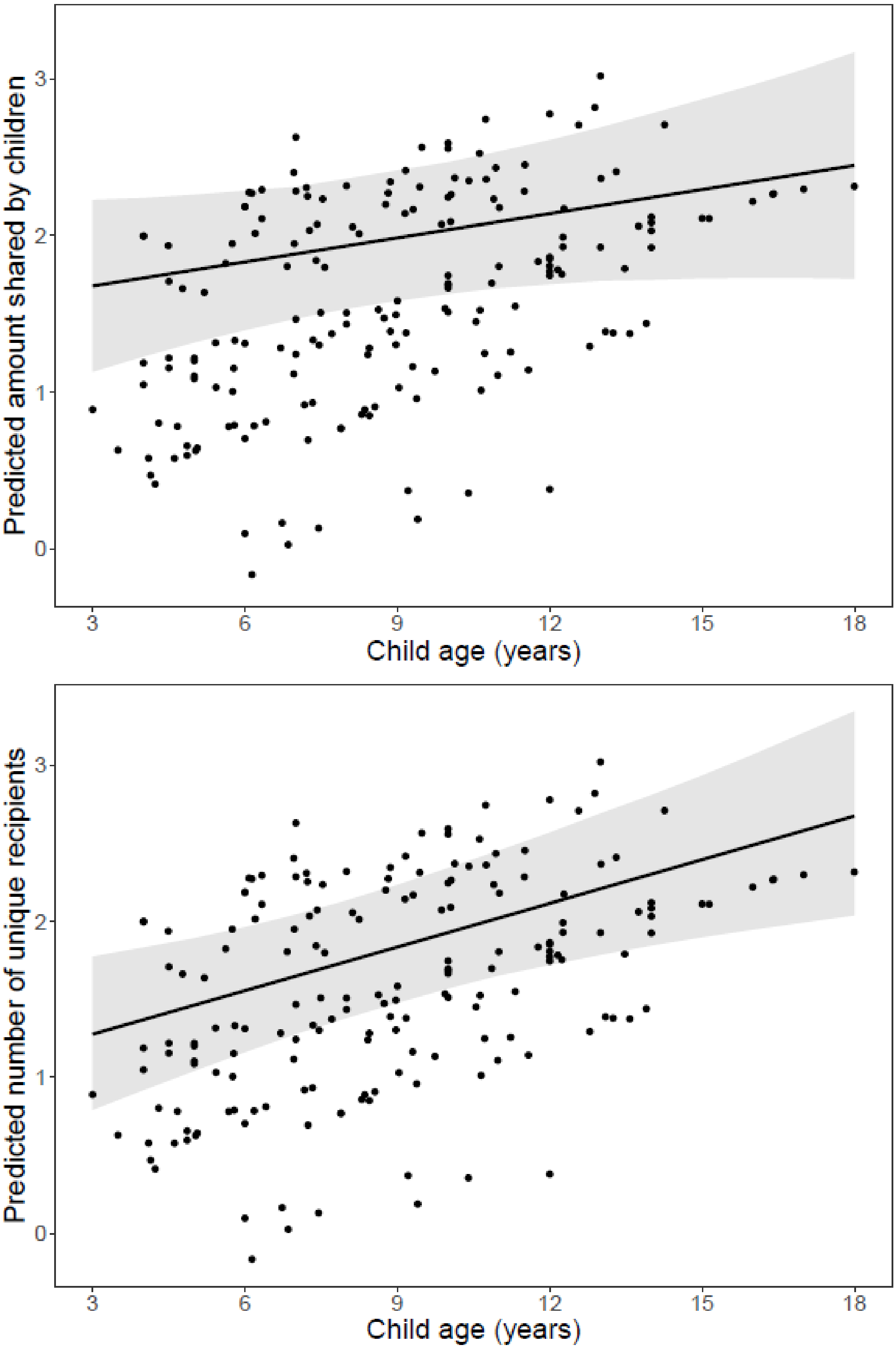

Fig S5: Scatterplots displaying the relationship between age and both overall levels of cooperation (total number of resources shared; upper panel); and the number of unique recipients that each child gave to (lower panel; *n*=179; camps=14). Points are based the predicted values from the parameters derived from the linear multivariable model on the respective outcome variable. The regression lines display the respective predictive values for each model, with model covariates set to their mean values (95% credible intervals in grey). There is only a weak relationship with age for total amount shared (upper), while a stronger positive relationship emerges for number of unique recipients (lower).

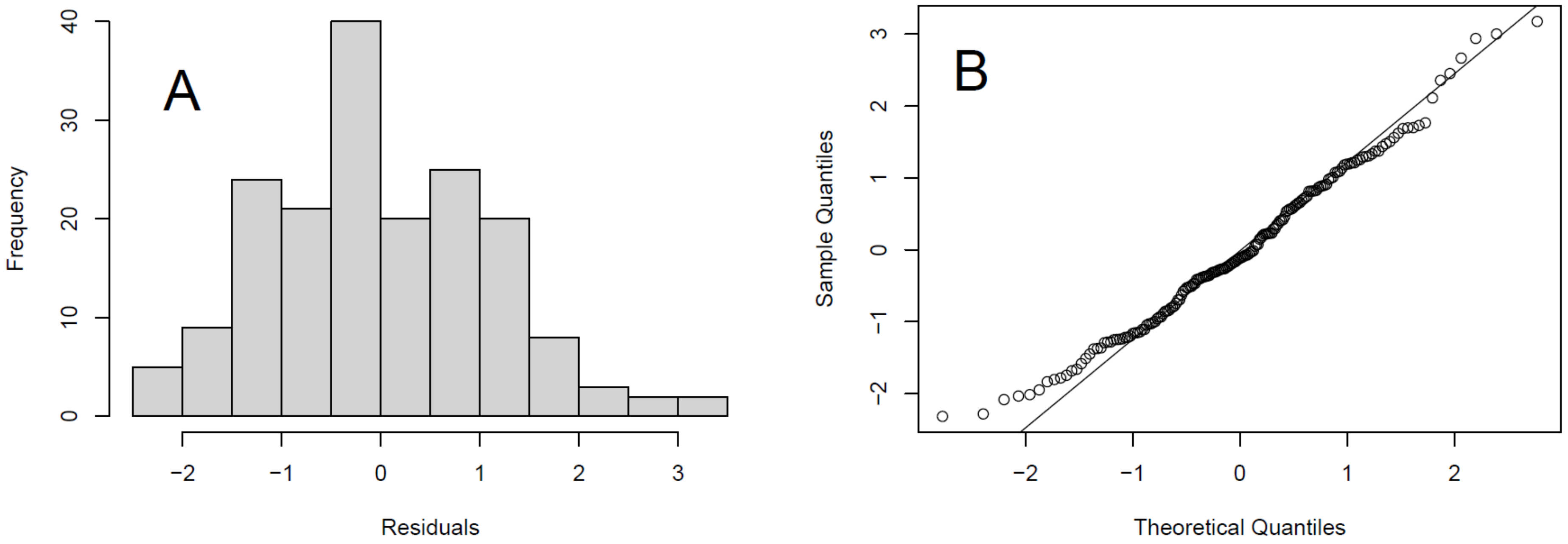

Fig S6: Testing the assumption of normality of the linear multivariable multi-level model with ‘number of unique recipients’ as the response variable, age, sex, average relatedness to child camp-mates, and average adult level of cooperation in camp as fixed effects and camp as a random effect. Both the histogram (A) and the Q-Q plot (B) indicate that the residuals of this models are approximately normally distributed (other than for more extreme values), meaning that the assumption of normality is unlikely to be violated substantially. Sensitivity analyses using multi-level Poisson and ordinal regression methods (which do not rely on assumptions of normality) find equivalent patterns of results to those of the linear multi-level model, suggesting that the conclusions reported in the main text are robust (Table S4).

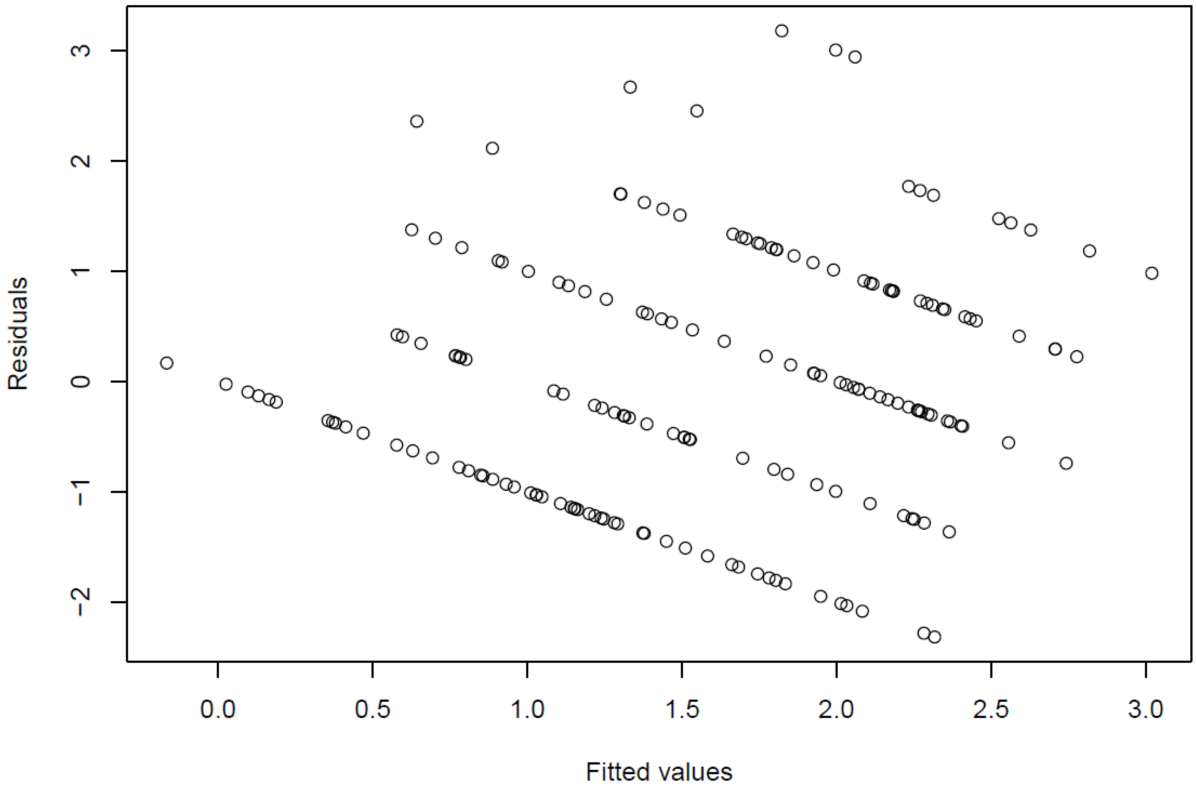

Fig S7: Testing the assumption of homoskedasticity of the linear multivariable multi-level model with ‘number of unique recipients’ as the response variable, age, sex, average relatedness to child camp-mates, and average adult level of cooperation in camp as fixed effects and camp as a random effect. This plot compares the residuals of this multivariable model against the fitted values. No strong ‘fan shape’ effects are apparent, meaning that the variance in residuals is approximately uniform for all fitted values (other than for extreme fitted values; below 0.5 and above 2.5, where the residual variance does appear to be lower). Nonetheless, given that the raw data for ‘number of unique recipients’ could only take on six possible values (between 0 and 5), the interpretation of this plot regarding the assumption of homoskedasticity is equivocal. However, sensitivity analyses using multi-level Poisson and ordinal regression methods (which do not rely on assumptions of homoskedasticity) find equivalent patterns of results to those of the linear multi-level model, suggesting that the conclusions reported in the main text are robust (Table S4).

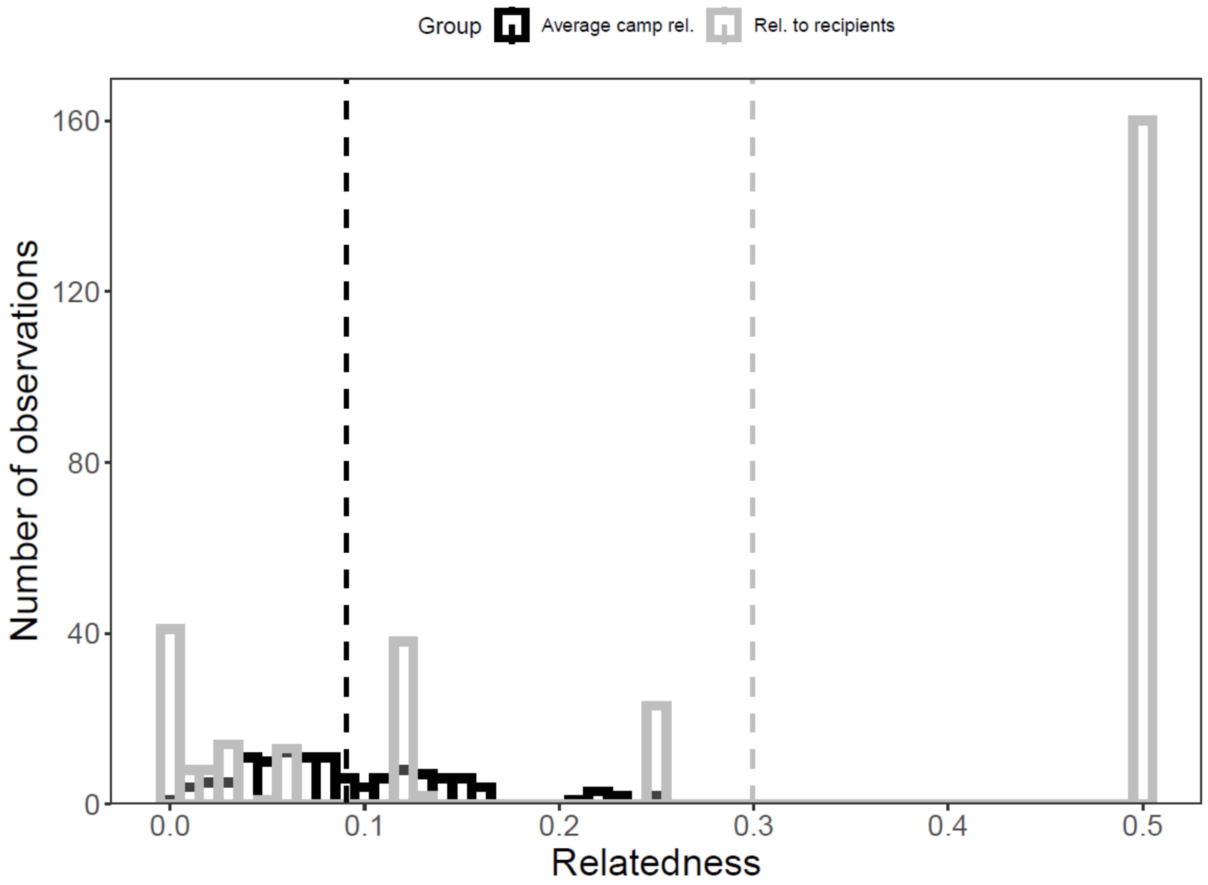

Fig S8: Histogram displaying: i) black bars: the average relatedness between the participant and all other children in the camp (*n*=125); and ii) grey bars: the relatedness between the participant and the recipient of the resource (*n*=308 gifts from 125 children). Dashed vertical lines indicate the mean relatedness values for each group.

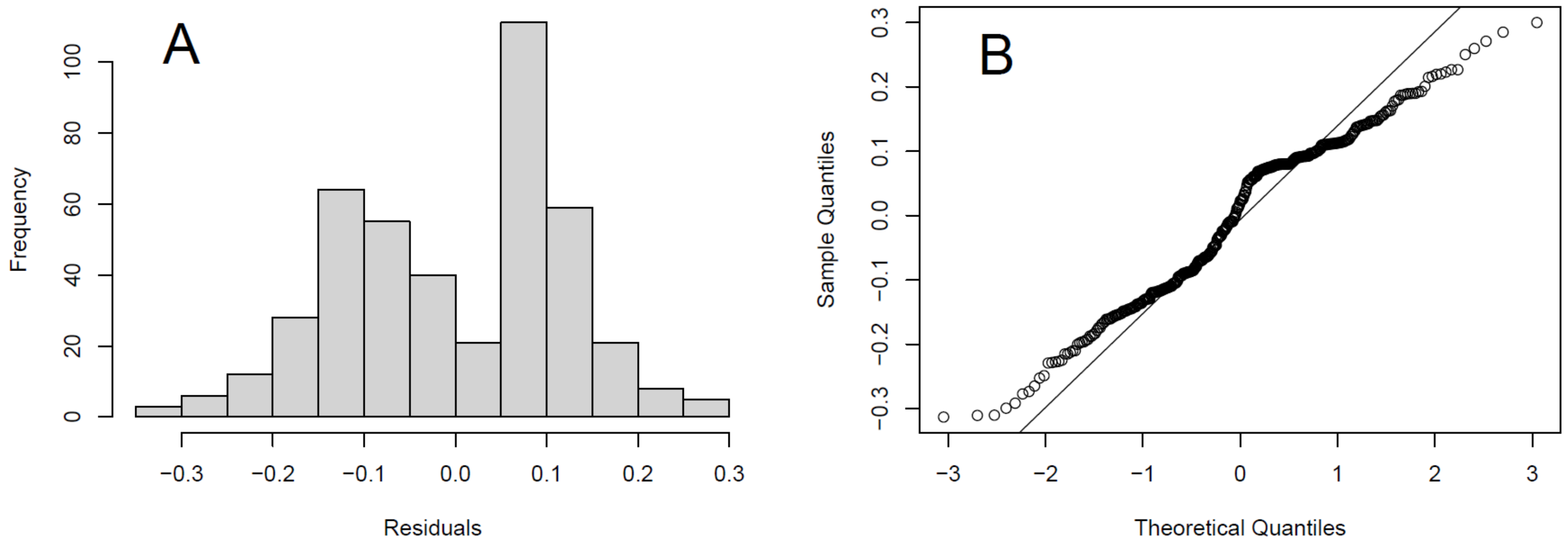

Fig S9: Testing the assumption of normality in the linear mixed-effects model assessing whether children were more likely to share with close kin, relative to background levels of camp relatedness to all children, with participant ID as a random effect to control for repeated nominations by the same individual. Both the histogram (A) and the Q-Q plot (B) indicate that the residuals of this models are definitely not normally distributed, violating the assumption of normality. However, as discussed in the main text, given that the magnitude of the effect is so large, this is unlikely to impact the general conclusion that children preferentially shared with close kin.

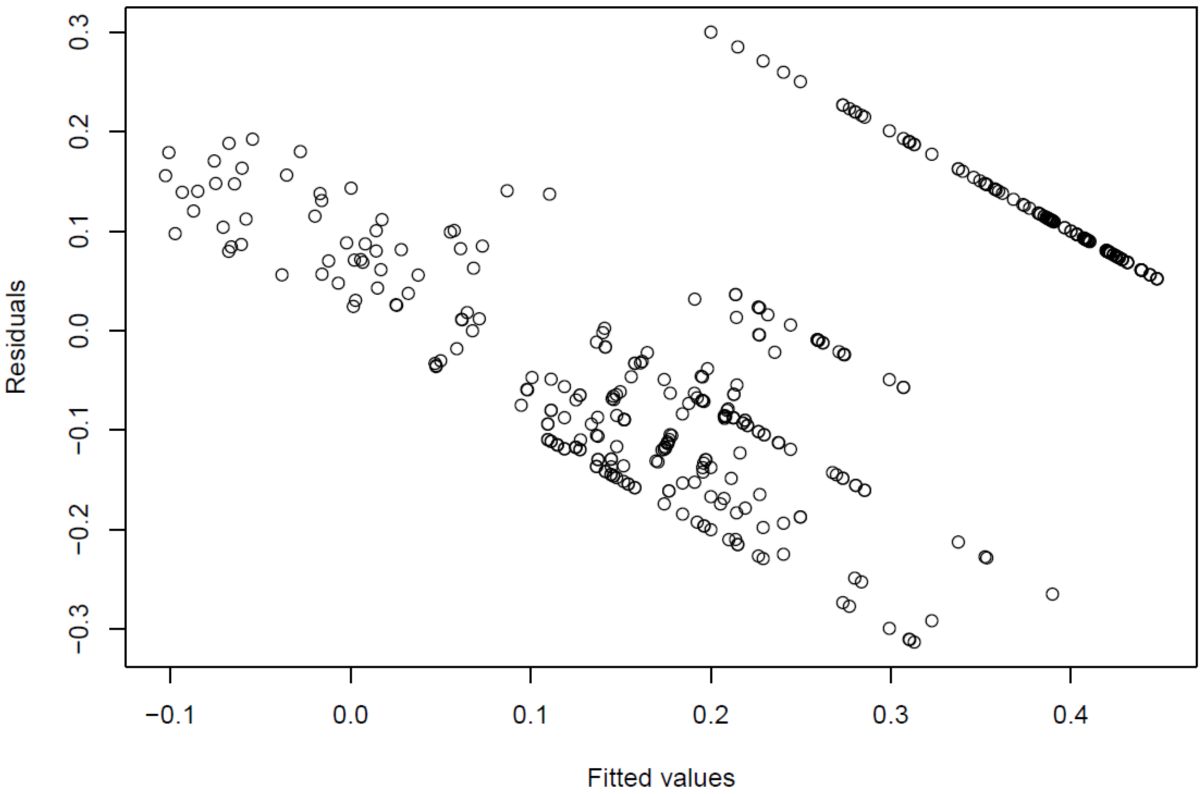

Fig S10: Testing the assumption of homoskedasticity in the linear mixed-effects model assessing whether children were more likely to share with close kin, relative to background levels of camp relatedness to all children, with participant ID as a random effect to control for repeated nominations by the same individual. This plot compares the residuals of this model against the fitted values. A 'fan shape' effect is apparent, with lower fitted values possessing less variance than greater values, meaning that the variance in residuals is not uniform for all fitted values. The assumption of homoskedasticity is therefore likely to be violated. However, as discussed in the main text, given that the magnitude of the effect is so large, this is unlikely to impact the general conclusion that children preferentially shared with close kin.

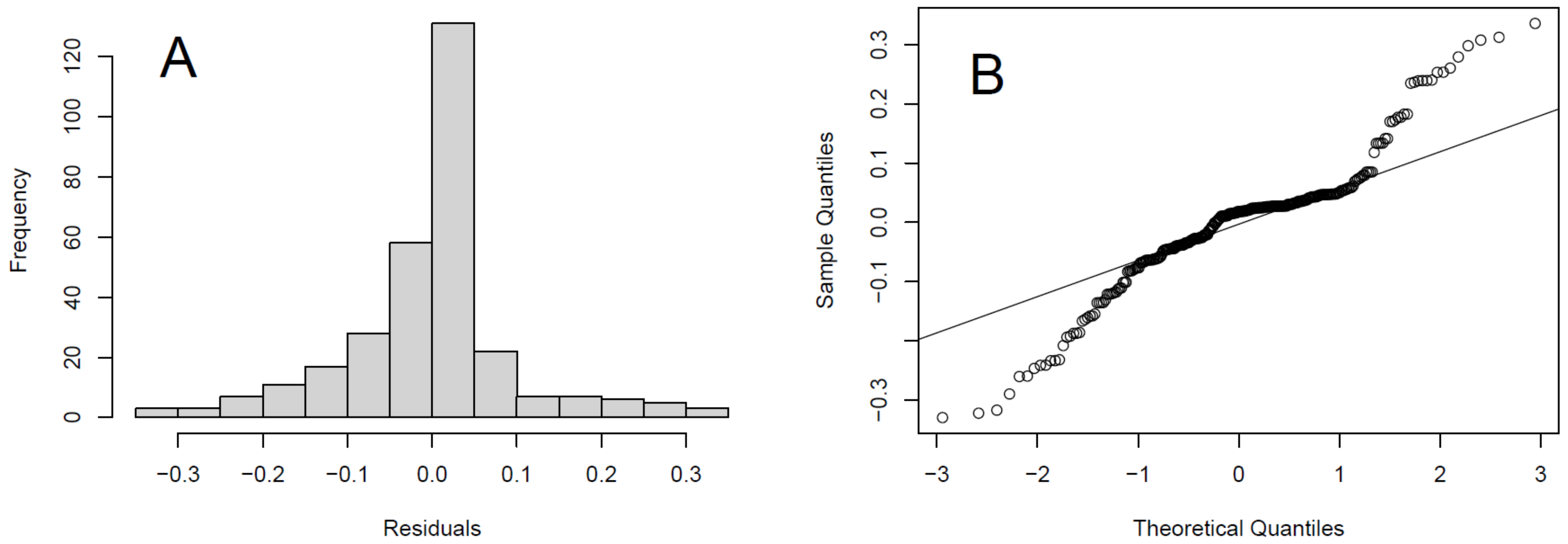

Fig S11: Testing the assumption of normality in the linear mixed-effects model assessing whether the relatedness between donor and recipient varied with the participant's age or sex, with participant ID as a random effect to control for repeated nominations by the same individual. Residual values are taken from the model with fixed effects of age and sex. Both the histogram (A) and the Q-Q plot (B) indicate that the residuals of this models are definitely not normally distributed, violating the assumption of normality. As a sensitivity check of the results presented in the main text, additional models are described in Table S5 which find qualitatively similar results, suggesting that these findings are robust to alternative model specifications.

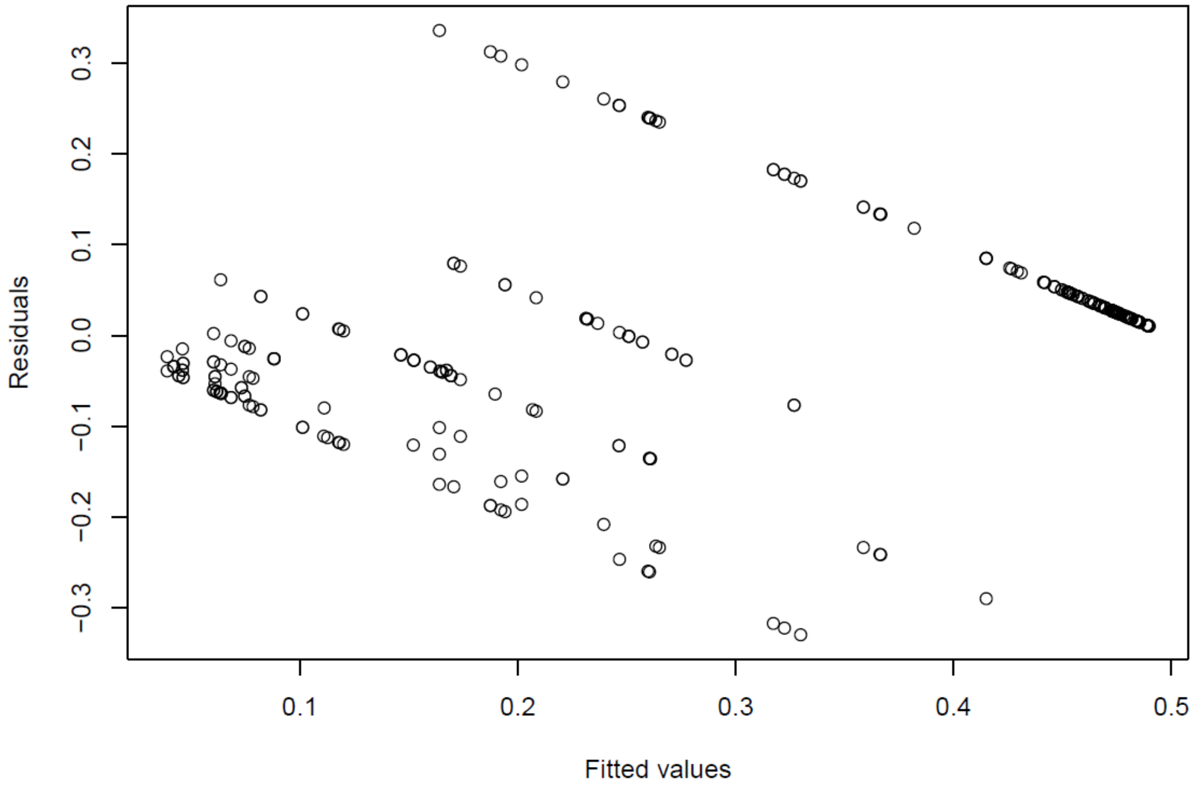

Fig S12: Testing the assumption of homoskedasticity in the linear mixed-effects model assessing whether the relatedness between donor and recipient varied with the participant's age or sex, with participant ID as a random effect to control for repeated nominations by the same individual. Residual values are taken from the model with fixed effects of age and sex. This plot compares the residuals of this model against the fitted values. While a 'fan shape' effect is not obviously apparent (other than at the extremes of the fitted values, where there is lower variance in the residuals), given the skewed distribution of relatedness data (Fig S8) the interpretation of this plot regarding the assumption of homoskedasticity is equivocal. As a sensitivity check of the results presented in the main text, additional models are described in Table S5 which find qualitatively similar results, suggesting that these findings are robust to alternative model specifications.

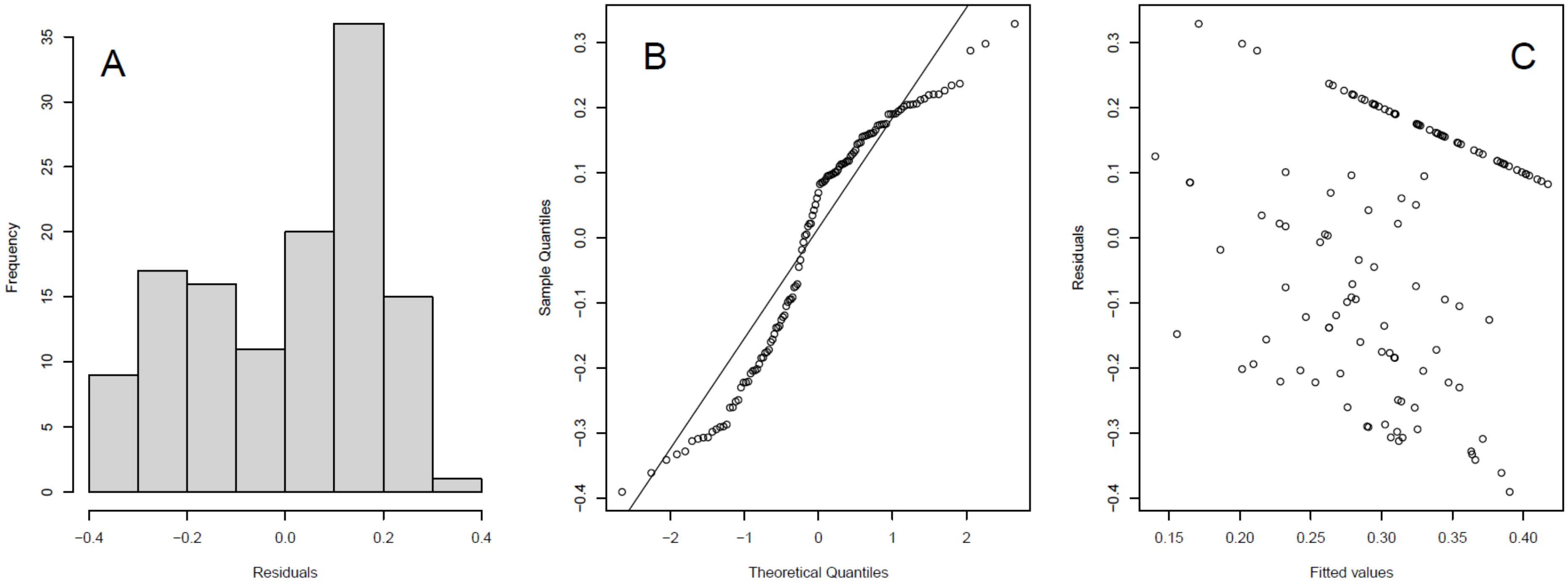

Fig S13: Testing the assumptions of normality (plots A and B) and homoskedasticity (plot C) in the single level model assessing whether the relatedness between donor and recipient varied with the participant's age or sex (see Table S5 for more details). Residual values are taken from the model with both fixed effects of age and sex. Both the histogram (A) and the Q-Q plot (B) indicate that the residuals of this models are definitely not normally distributed, violating the assumption of normality. In plot C there is little obvious patterning of residuals by their fitted values, suggesting that the assumption of equal variances may be met in this model.

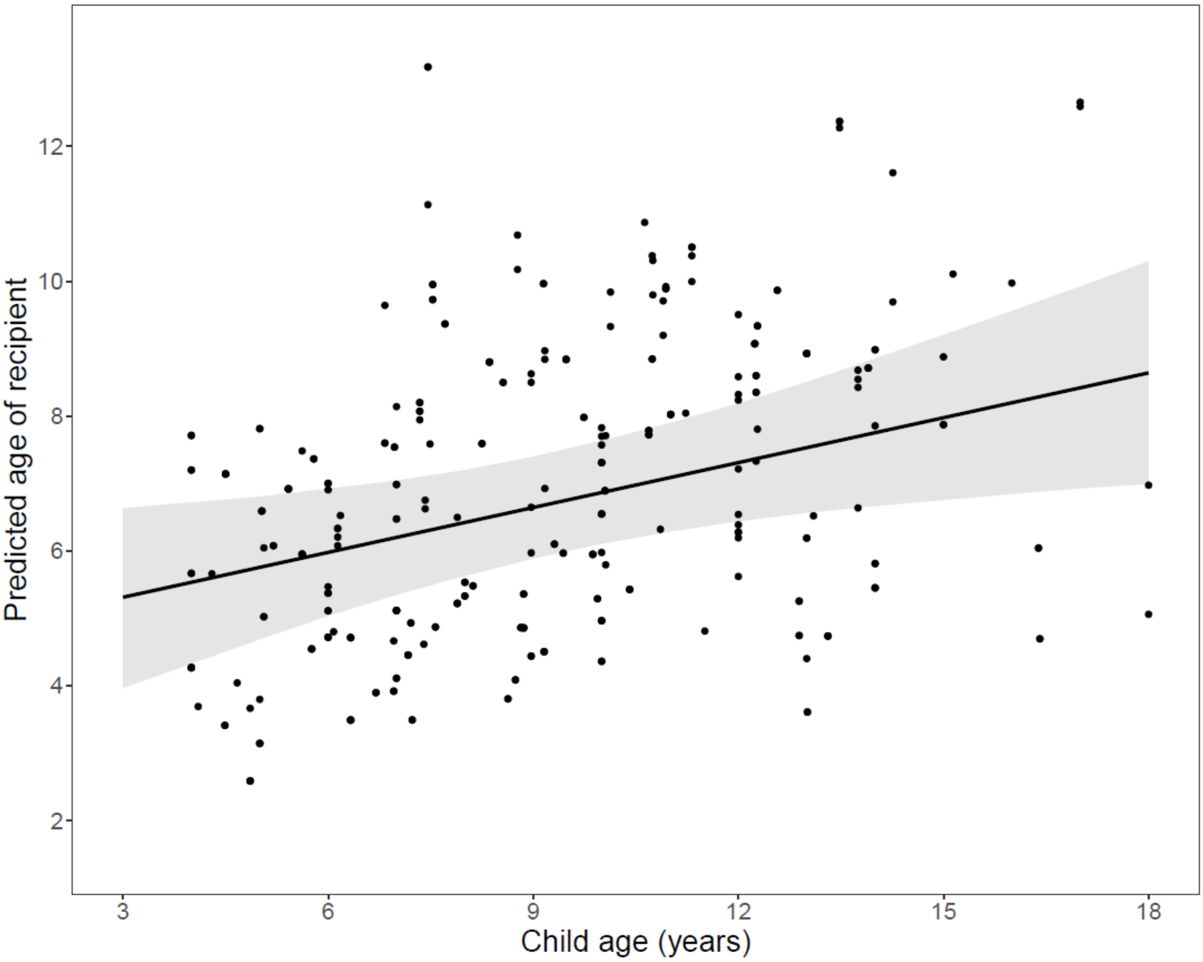

Fig S14: Scatterplot of the relationship between child age and the predicted age of recipient based on the model in Table S6 (*n*=308 gifts from 125 children). The regression line displays the predicted age of recipient for a range of age values from said model, with sex set at 0.5 (95% credible interval in grey).

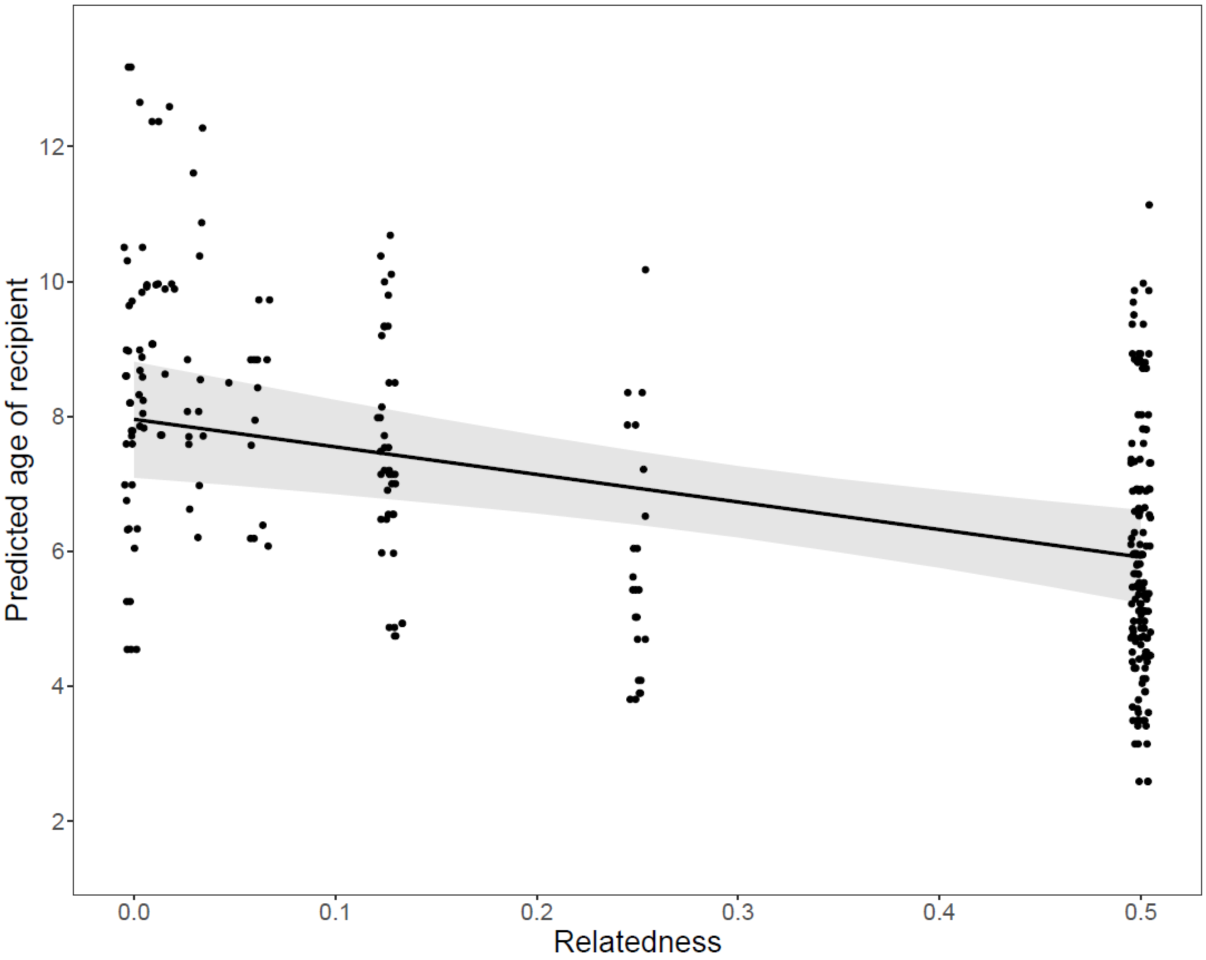

Fig S15: Scatterplot of the relationship between relatedness of participant to recipient and the predicted age of recipient based on the model in Table S6 (*n*=308 gifts from 125 children). The regression line displays the predicted age of recipient for a range of relatedness values from said model, with sex set at 0.5 (95% credible interval in grey). Jitter has been added to this plot to avoid overlapping data points.

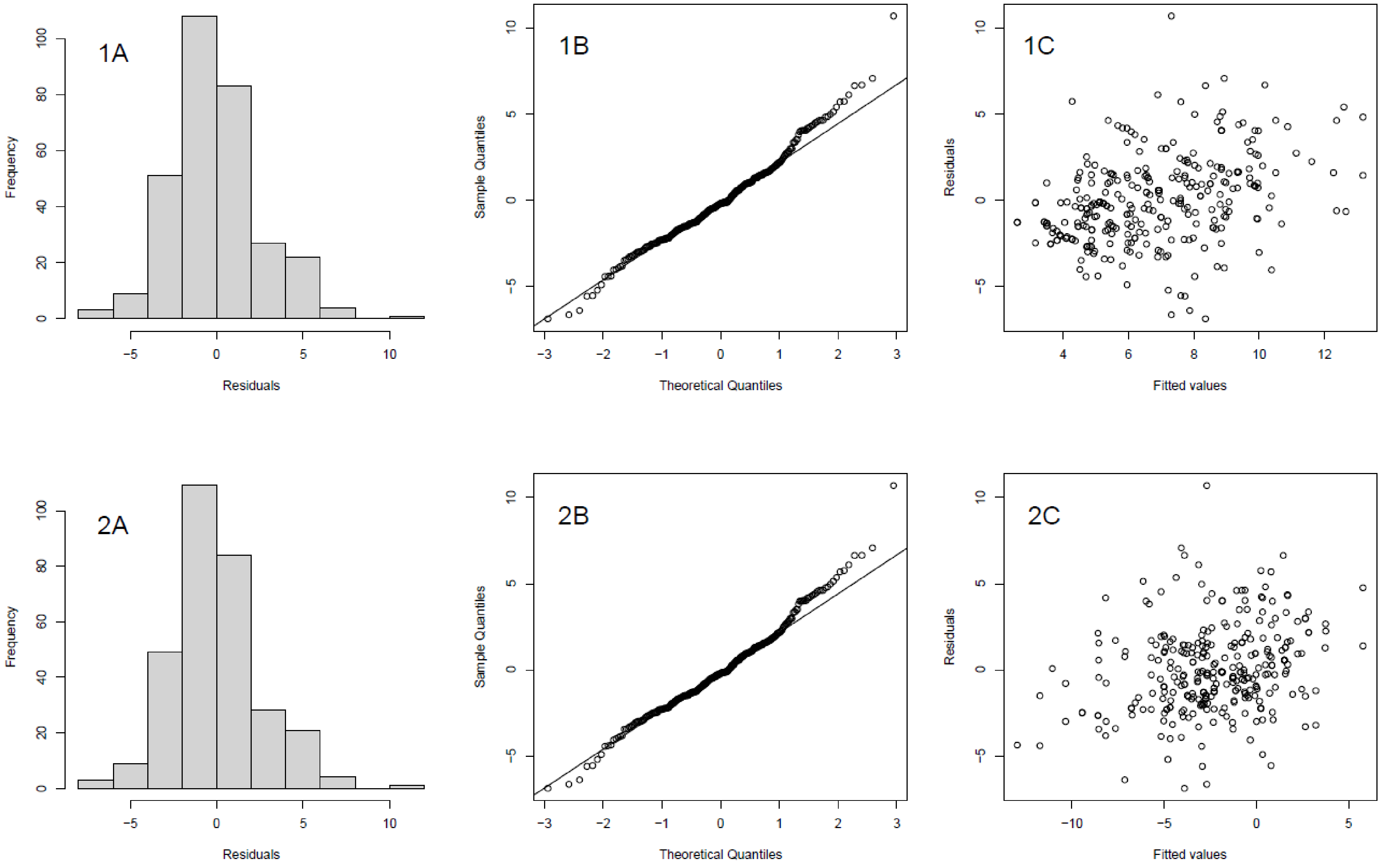
1A
Frequency
Residuals
1B
Sample Quantiles
Theoretical Quantiles
1C
Residuals
Fitted values
2A
Frequency
Residuals
2B
Sample Quantiles
Theoretical Quantiles
2C
Residuals
Fitted values

Fig S16 (previous page): Testing the assumptions of normality (plots 1A, 1B, 2A and 2B) and homoskedasticity (plots 1C and 2C) in the linear mixed-effect models assessing whether participant age, participant sex and relatedness were associated with recipient age (row 1; Table S6) and the age difference between participant and recipient (row 2; Table S7). Residual values are taken from the models which include fixed effects of participant age, participant sex, relatedness. Both the histograms (1A and 2A) and the Q-Q plots (1B and 2B) indicate that the residuals of this models are approximately normally distributed. Note that the residuals from both these models are practically identical; this is because the recipient age model (Table S6) and the age difference model (Table S7) are essentially measuring the same thing, given that recipient age is simply the age difference plus participant's age (and hence why the parameter estimates of the fixed effects in Tables S6 and S7 are similar for sex and relatedness terms). In plots 1C and 2C there is no obvious patterning of residuals by their fitted values, suggesting that the assumption of equal variances may be met in these models.
