# Supplementary material for: Cooperation and partner choice among Agta hunter-gatherer children: An evolutionary developmental perspective
Source: PLoS One. 2023 Apr 26;18(4):e0284360. doi: 10.1371/journal.pone.0284360 (PMC10132543; doi:10.1371/journal.pone.0284360)
Supplement: Attachment — Submitted filename: Comments.pdf [file pone.0284360.s002.pdf]

Review for PLoS One:

Cooperation and Partner Choice Among Agta Hunter-Gatherer Children: An Evolutionary Developmental Perspective

Summary

My overall impression is that this article will constitute a publishable—and valuable—piece of scholarship. The topic is interesting, and the data seem great. The authors also have a delightful and refreshing writing style, where they make the potential flaws/inferential concerns of their work clear and visible, and comment on how such issues might be resolved in future work. This is commendable, and heightens the trust that readers have in the credibility of the authors, especially at a time when over-statement is so common.

In terms of the overall writing, I think the paper is good, and probably falls within the standards of quality expected by PLoS. It could probably be accepted with minor revisions. However, with slightly more extensive revisions, the paper could be made significantly stronger. For example, the entire paper is based around analysis of network data, and the authors even cite a textbook [1] with a fully worked out example of how to analyze such network data (using the Social Relations Model), but they don't make use of network analysis techniques. Given the fact that network analysis is a big deal these days, and the fact that tools are already out there, this comes across as a pretty big oversight.

I think the authors should consider making some changes, as I outline below, as they should significantly improve the methodological strength of the paper, and allow the authors to remove many of the wayward sentences about how certain statistical analyses couldn't be done because of "convergence problems" and "non-nonsensical" results.

Minor Comments

- Lines 80:100—the authors talk a bit about how past experimental studies fail to account for partner choice when deploying experimental economic games. It might be worth pointing out, that there are a lot of people in the field working on deploying similar kinds of network-structured games: e.g., Rucas et al. [2], Gervais [3], Pisor et al. [4], and Redhead et al. [5] to study cooperation and partner choice. The author's study differs from these papers, in that their methods target children's cooperative networks, but the methods have an intellectual history that could be referenced, and work has been done on validating such network-based study designs against other forms of network data [e.g., 4].
- Lines 548:563—A similar point comes up again in the discussion. The authors talk about the limitations of the study, and reference the use of economic games as "chief among such limitations". Acknowledging that games may have limited validity is good practice, but I think the authors might go a bit to far here. I think games based on behavior towards imaginary "anonymous" others are highly suspect, but I think using dyadic experimental game data (as was done here) goes a long way towards addressing these concerns. When people can make decisions based on real socio-relational information, games become a lot more reliable. Again, see: Gervais [3] and Pisor et al. [4] for some discussion to offset the concerns raised in Gurven and Winking [6].
- Lines 244:250—I was taught in grad school almost 10 years ago that `lme4` was out-dated, and that it not possible to fit multi-level model accurately without using MCMC. Nowadays, people use `Stan` to fit these kinds of models. There are code templates everywhere, and `R` packages like `rethinking` and `brms` that can automatically build and run `Stan` models using simple, base-R notation.
- Adopt statistical methods that are appropriate for analyzing network data (e.g., see the Social Relations Model [1, 7] for dealing with sender, receiver, and dyadic effects in a unified model).

Good work so far, and best wishes on the manuscript.

References

[1] Richard McElreath. *Statistical rethinking: A Bayesian course with examples in R and Stan*. CRC press, 2020.

[2] Stacey L Rucas, Michael Gurven, Hillard Kaplan, and Jeffrey Winking. The social strategy game. *Human Nature*, 21(1):1–18, 2010.

[3] Matthew M Gervais. Rich economic games for networked relationships and communities: development and preliminary validation in yasawa, fiji. *Field methods*, 29(2):113–129, 2017.

[4] Anne C Pisor, Matthew M Gervais, Benjamin G Purzycki, and Cody T Ross. Preferences and constraints: the value of economic games for studying human behaviour. *Royal Society open science*, 7(6):192090.

[5] Daniel Redhead, Augusto Dalla Ragione, and Cody T. Ross. Friendship and partner choice in rural colombia. *Evolution and Human Behavior*, 2022. ISSN 1090-5138. doi: https://doi.org/10.1016/j.evolhumbehav.2022.08.004. URL https://www.sciencedirect.com/science/article/pii/S1090513822000484.

[6] Michael Gurven and Jeffrey Winking. Collective action in action: Prosocial behavior in and out of the laboratory. *American Anthropologist*, 110(2):179–190, 2008.

[7] Jeremy M Koster and George Leckie. Food sharing networks in lowland Nicaragua: An application of the social relations model to count data. *Social Networks*, 38:100–110, 2014.
