# Supplementary material for: Cooperation and partner choice among Agta hunter-gatherer children: An evolutionary developmental perspective
Source: PLoS One. 2023 Apr 26;18(4):e0284360. doi: 10.1371/journal.pone.0284360 (PMC10132543; doi:10.1371/journal.pone.0284360)
Supplement: Attachment — Submitted filename: KidsCoopPaper_ResponseToReviewers.pdf [file pone.0284360.s003.pdf]

Cooperation and partner choice among Agta hunter-gatherer children: an evolutionary developmental perspective (PONE-D-22-29549) – Reviewer 1

Reviewer 1 comments:

Summary

My overall impression is that this article will constitute a publishable and valuable piece of scholarship. The topic is interesting, and the data seem great. The authors also have a delightful and refreshing writing style, where they make the potential flaws/inferential concerns of their work clear and visible, and comment on how such issues might be resolved in future work. This is commendable, and heightens the trust that readers have in the credibility of the authors, especially at a time when over-statement is so common.

In terms of the overall writing, I think the paper is good, and probably falls within the standards of quality expected by PLoS. It could probably be accepted with minor revisions. However, with slightly more extensive revisions, the paper could be made significantly stronger. For example, the entire paper is based around analysis of network data, and the authors even cite a textbook [1] with a fully worked out example of how to analyze such network data (using the Social Relations Model), but they don't make use of network analysis techniques. Given the fact that network analysis is a big deal these days, and the fact that tools are already out there, this comes across as a pretty big oversight.

I think the authors should consider making some changes, as I outline below, as they should significantly improve the methodological strength of the paper, and allow the authors to remove many of the wayward sentences about how certain statistical analyses couldn't be done because of “convergence problems" and “non-nonsensical" results.

We thank the reviewer for their helpful and constructive comments, and appreciate the overall positive impression of our paper. We hope that our revised manuscript and responses to the reviewer’s comments have sufficiently addressed the reviewer’s concerns. The reviewer’s original comments are in black font and our responses in blue.

Regarding the use of network methods, we certainly see the merit in taking a network approach to analysing data similar to these. However, for our data we do not believe that a network analysis would be appropriate. This is predominantly because network analyses, such as the Social Relations Model, require a full network (i.e., everyone could be a potential giver and receiver). In our data, many of the recipients did not participate in these games, either because they were too young – many recipients were infants – or simply chose not to take part. To the best of my knowledge, such network analyses would not be possible with these data. Given this, we focused our sharing partners analyses using each gift as a data point, with random effects to control for dependencies within the data (i.e., repeated nominations by the same child). We believe that the current methods – while perhaps somewhat piecemeal and less elegant than a unified network analysis – are suitable for answering the questions set out in our paper, given the data available. We have added

text to this effect justifying our choice of analysis in the manuscript (lines 322-338): “This approach was chosen over a network analysis, such as the Social Relations Model (48,50), predominantly because many of the recipients did not participate in the games, either because they were too young – many recipients were infants – or chose not to take part. Given this data structure, such network analyses would not be possible.”.

Minor Comments

- Lines 80:100 - the authors talk a bit about how past experimental studies fail to account for partner choice when deploying experimental economic games. It might be worth pointing out, that there are a lot of people in the field working on deploying similar kinds of network-structured games: e.g., Rucas et al. [2], Gervais [3], Pisor et al. [4], and Redhead et al. [5] to study cooperation and partner choice. The author's study differs from these papers, in that their methods target children's cooperative networks, but the methods have an intellectual history that could be referenced, and work has been done on validating such network-based study designs against other forms of network data [e.g., 4].
  Thanks for this suggestion. We have now added a sentence on this in the introduction (lines 139-142): “While experimental designs which incorporate partner choice are increasingly employed among adults (24,36–39), there are few studies applying such methods with children, especially in small-scale societies.”

- Lines 548:563 - A similar point comes up again in the discussion. The authors talk about the limitations of the study, and reference the use of economic games as “chief among such limitations". Acknowledging that games may have limited validity is good practice, but I think the authors might go a bit to far here. I think games based on behavior towards imaginary “anonymous" others are highly suspect, but I think using dyadic experimental game data (as was done here) goes a long way towards addressing these concerns. When people can make decisions based on real socio-relational information, games become a lot more reliable. Again, see: Gervais [3] and Pisor et al. [4] for some discussion to offset the concerns raised in Gurven and Winking [6].
  Yes, good point, we agree (and this was one of the motivations for using such non-anonymous methods). A sentence on this has now been added to the discussion (lines 594-597): “Additionally, many criticisms about the validity of experimental games apply to methods with anonymous recipients; by employing methods with non-anonymous recipients here, it is hoped that many of these concerns will be addressed and results more reliable (37,38).”

- Lines 244:250 - I was taught in grad school almost 10 years ago that lme4 was out-dated, and that it not possible to fit multi-level model accurately without using MCMC. Nowadays, people use Stan to fit these kinds of models. There are code templates everywhere, and R packages like rethinking and brms that can automatically build and run Stan models using simple, base-R notation.

Given the feedback by both reviewers on this point we have now re-done all of the analyses using Bayesian methods (using the *brms* package). While the overall pattern of results is the broadly the same, there are fewer convergence issues and non-sensical results, and we believe that the results are now stronger and conclusions more robust. The methods and results sections have been updated accordingly.

- Adopt statistical methods that are appropriate for analyzing network data (e.g., see the Social Relations Model [1, 7] for dealing with sender, receiver, and dyadic effects in a unified model).
  Please see the justification above for why these network methods would not be feasible here.

Good work so far, and best wishes on the manuscript.

References

[1] Richard McElreath. Statistical rethinking: A Bayesian course with examples in R and Stan. CRC press, 2020.

[2] Stacey L Rucas, Michael Gurven, Hillard Kaplan, and Jeffrey Winking. The social strategy game. Human Nature, 21(1):1-18, 2010.

[3] Matthew M Gervais. Rich economic games for networked relationships and communities: development and preliminary validation in yasawa, Fiji. Field methods, 29(2):113-129, 2017.

[4] Anne C Pisor, Matthew M Gervais, Benjamin G Purzycki, and Cody T Ross. Preferences and constraints: the value of economic games for studying human behaviour. Royal Society open science, 7(6):192090.

[5] Daniel Redhead, Augusto Dalla Ragione, and Cody T. Ross. Friendship and partner choice in rural colombia. Evolution and Human Behavior, 2022. ISSN 1090-5138. doi: https://doi.org/10.1016/j.evolhumbehav.2022.08.004. URL https://www.sciencedirect.com/science/article/pii/S1090513822000484.

[6] Michael Gurven and Jeffrey Winking. Collective action in action: Prosocial behavior in and out of the laboratory. American Anthropologist, 110(2):179-190, 2008.

[7] Jeremy M Koster and George Leckie. Food sharing networks in lowland Nicaragua: An application of the social relations model to count data. Social Networks, 38:100-110, 2014.

Cooperation and partner choice among Agta hunter-gatherer children: an evolutionary developmental perspective (PONE-D-22-29549) – Reviewer 2

Reviewer 2 comments:

This is an interesting paper that investigates the level and pattern of cooperative behaviours of Agta children over the developmental period, using a simple resource allocation game. Although many other experimental studies have explored how cooperative behaviours emerge in children and increase over childhood, this study provides additional details and identifies with whom children are likely to share. Surprisingly, only the average level of cooperation among adults in camp had the effect on the level of cooperation of children. Age, sex, or within-camp average relatedness did not have any effects how much children shared with others. Regarding sharing partners, children were more likely to share with close kin, and children increasingly shared with less-related individuals as they aged.

This work presents an experimental dataset investigating how much and with whom children share the given resources in a hunter-gatherer population. The result supports one of well-known propensities of human cooperation: the tendency to increase the benefit of close relations, and further provides an evidence that children's cooperative networks increases with age, from kin to distant kin and non-kin. I believe that it is potentially a very important and insightful paper.

However, there are a few points which should be addressed before further assessment. I have a few main concerns with this paper. First, the predictions are not clearly justified theoretically especially in the Introduction, some of them were discussed later only in Discussion. Second, the authors need to be more careful when interpreting the results and formulating sentences throughout the paper—specifically about partner choice. Third, the paper needs to be re-organised to be clear in explaining the statistical analysis methods and results, as with the current state it is hard to follow what the authors did for statistical analyses and, thus, it is hard to draw the main findings and interpretation the authors claimed that they found. Lastly and most importantly, the authors should use alternative methods to solve several convergence issues that they faced during analyses. I also believe that the paper needs substantial revision on their writing. to See more details 'Major issues' below.

We thank the reviewer for their helpful and constructive comments. We hope that our revised manuscript and responses to the reviewer's comments have sufficiently addressed the reviewer's concerns. The reviewer's original comments are in black font and our responses in blue.

Major Issues

1. This paper aims to investigate cooperative behaviours of children from developmental perspectives, but the predictions do not clearly provide theoretical reasoning for these

predictions from developmental perspectives. The main predictions are that the level of cooperation would increase with age (L 129) and that children's level of cooperation would vary between camps and will correspond to the adults' patterns of cooperation in each camp (L 130-133). In the current text, it reads like the authors set the predictions because previous research found the same pattern, and therefore they also expect to find the same pattern. Explaining clear reasoning for predictions which stands on theory in the Introduction will make this paper interesting. Developmental research has demonstrated that children are sensitive to social norms, and that and that social norms are internalised by middle childhood. Social learning research has shown that that social learning mechanisms contribute to children's acquisition of community-specific social norms, including sharing norms. This study can add to this body of research by investigating developmental trajectories of sharing behaviours. However, social learning is discussed only later in the Discussion and social norms are not mentioned. I think it is important to bring theory in Introduction when building the predictions.

We have provided additional theoretical justification to support these predictions in the introduction (lines 78-80): "One explanation for this pattern of results is that middle childhood may be a key time when children become more sensitive to the local social norms and alter their cooperative behaviour accordingly (11,14)." Note that we have kept this theoretical exposition relatively brief to try and avoid increasing the length of the paper too much.

2. The second research question and prediction the authors are investigating is how partner choice shapes children's cooperative behaviour (L 90, L 98). However, this work did not explore how partner choice shapes cooperation, but is only examining who are sharing partners and how this change over childhood.

Apologies if we were not clear enough here in our original submission. The inclusion of partner choice in these experimental games is important for two reasons. First, forcing children to cooperate with certain partners (as per most previous developmental studies) may bias assessments of overall levels of cooperation. That is, partner choice may shape overall levels of cooperation (if older children are more likely to share with strangers or non-kin, for instance). The second reason for exploring partner choice is – as the reviewer says – to explore sharing partners and how these change over childhood.

We have expanded and restructured the introduction to make these points clearer (lines 84-142):

"However, the majority of these studies have used experimental methods which either paired children with anonymous others (e.g., (10,14)) or with unrelated individuals (although partners were known to each other, such as attending the same school or living in the same village; e.g., (11,12,15)). These studies may overlook the important role of 'partner choice' – who children choose to interact and cooperate with – in determining cooperative behaviour (16). This may be important for two reasons. First, who children are interacting with is likely to impact how much they cooperate, meaning that ignoring the role of partner

choice may confound assessments of overall levels of cooperation. For instance, say that young children preferentially only share with people they know, while older children are more likely to share with strangers. In experimental games with anonymous recipients, older children may appear more cooperative than younger children, but this may be due to partners being anonymous, rather than differences in overall levels of cooperation. By only assessing cooperation towards non-kin or strangers, many previous studies may have overlooked how partner choice shapes children's cooperative behaviour.

A second reason for considering partner choice is that it allows an exploration of who children share with and whether this changes over time. […]

Incorporating partner choice into experimental games with children is therefore necessary for two reasons: i) to allow comparisons of overall levels of cooperation which are not confounded by constraining children to certain partners; and ii) to allow an exploration of who children share with and whether this changes with age. While experimental designs which incorporate partner choice are increasingly employed among adults (24,36–39), there are few studies applying such methods with children, especially in small-scale societies."

3. The Method and Result sections need quite a bit of re-organisation to make the paper clearer. The results section is hard to follow because the explanations on models are mixed and the organisation is repetitious, thus, hard to understand what the main findings are and to draw the interpretation. Instead of repeating the model structures in the Results section, I suggest the authors to explain statistical model structures more clearly in the Methods part, probably by using a table with model structures or by bringing a DAG figure to the main text (which is only in Supplementary Information), and to start the Results with clear descriptions of their findings. For example, model explanations (L 326 – L 333, L 403 – 409, L 427 – 430, L 436 – L440, L441 – 444) in the Results can be moved into the statistical analysis part and deleted from here.

We have now re-organised the methods and results section to try and improve clarity. All of the models are now explained in the 'Statistical analysis' section of the Methods, which we hope simplifies the Results section and makes the key findings clearer to readers.

4. My biggest concern regards the statistical analysis methods. The authors faced several convergence issues during statistical analyses and they noted that therefore they used similar models (L 249, L 299, L 367, L 604). Yet there are many analysing methods which can solve convergence issues, for example, by using Bayesian modelling (also noted by the authors in Line 607). I wonder why the authors would not use Bayesian modelling, for example, using 'brms' package in R. Moreover, in Line 424 and Line 438, the authors wrote that the assumptions of normality and homoskedasticity were violated due to highly skewed distribution of the data. Here I am not convinced how this is not a problem (L 425) and whether those model results can be interpreted as it is in the paper now.

Given the feedback by both reviewers on this point we have now re-done all of the analyses using Bayesian methods (using the *brms* package). While the overall pattern of results is the broadly the same, there are fewer convergence issues/failed models, and we believe that the results are now stronger and conclusions more robust. The methods and results sections have been updated accordingly.

Re. assumptions of normality and homoskedasticity, we have added a couple of sentences to the discussion justifying why these violations are unlikely to impact our interpretation (lines 645-649): "Additionally, although some models violated the assumptions of normality and homoskedasticity, these violations are unlikely to lead to biased effect estimates (63). Nonetheless, where possible, alternative model specifications, which do not rely on these assumptions, found qualitatively similar patterns of results, suggesting that these conclusions are robust."

5. The paper does not read clearly and smoothly. The authors need to increase clarity and brevity of the paper, while avoiding much repetition. Especially, the Discussion is very hard to follow and thus to buy their claims. For example, in Discussion the authors acknowledged the limitation of the study over several paragraphs. It is good that the authors brought up the limitation of the study, addressed their points, and suggested the future directions. However, those points were not addressed well in scientific writing style— for example, Line 548, "This study of course possesses limitations. Chief among these is that cooperation is measured via experimental games, meaning that is it not clear how this links to real-world cooperative behaviours." This sentence should be formulated again. Moreover, despite the valuable contribution of this study, the authors' voice became too week after explaining the limitation to buy their claims. This is clearly shown in the last sentence of the last paragraph of the paper (from Line 652), saying "While these specific conclusions are tentative and rather speculative, this study has hopefully demonstrated the potential benefits of taking an adaptive evolutionary approach to understanding the development of children's cooperative behaviour.", which is quite different from their voice in the last sentence of the first paragraph of the Discussion (Line 464-467), saying "Together, these results provide an insight into the evolutionary and developmental roots of cooperation among Agta hunter-gatherer children, and have implications for developmental studies of cooperation and the evolution of human childhood and life history more widely." With this varying tone and voice, it is hard for readers to believe the conclusion of this study. The paper needs substantial revision on their writing style.

This is a more difficult point to address, as the other reviewer said "The authors also have a delightful and refreshing writing style, where they make the potential flaws/inferential concerns of their work clear and visible, and comment on how such issues might be resolved in future work. This is commendable, and heightens the trust that readers have in the credibility of the authors, especially at a time when over-statement is so common." Given these conflicting assessments, opinions regarding the writing style appear rather subjective.

Nonetheless, we have taken onboard the points raised by the reviewer and re-phrased the paper accordingly. For instance, at the end of the discussion (line 676) we have changed "While these specific conclusions are tentative and rather speculative" to "While these specific conclusions are tentative and require replication". As this is just a single study on one population using a relatively novel methodology, we are hesitant about making grand sweeping claims, but do believe that these results are interesting and may have important implications for understanding the evolution and development of cooperative behaviour (specifically for the Agta, but hopefully more broadly as well). We also do not necessarily see the conflict regarding 'voice' in the first and last paragraphs; we feel that these results may help inform future work in this area (first paragraph of discussion), but that our theoretical interpretation of the results is tentative, requires further study, and that others may disagree (last paragraph of discussion).

We have also thoroughly edited the paper throughout to improve readability (e.g., shortening sentences, removing brackets).

Minor Issues

1. I would include 'sharing' explicitly throughout the text, so that the paper can read more clearly: for example, in title "Cooperation and sharing partners among…"

We have updated this in some places, but as partner choice can impact levels of cooperation in addition to simply referring to sharing partners (as discussed above), we have kept 'partner choice' where appropriate (including in the title).

2. Regarding partner choice, the authors can define it more clearly what it means in this study -- sharing partners.

This has been updated (lines 87-89): "These studies may overlook the important role of 'partner choice' – who children choose to interact and cooperate with – in determining cooperative behaviour (16)."

3. Throughout the manuscript, the authors can use brackets "( )" a lot to explain something. I suggest to avoid using brackets too much, and rather write it out as a full sentence.

We have cut down on the use of brackets throughout the manuscript.

4. In Ethnographic background, I suggest the authors to describe the structure of each camp, what varies (relatedness, number, sex ratio) and how it varies, as camp variation is one of the important factors influencing the results.

This additional information has been added to table S1. However, we have not discussed this in detail in the text as describing each camp will take a lot of space, and because camp-

level differences in age, relatedness and sex ratio are not especially relevant to the main analyses. The most important aspect of camp-level variation is in adult and children's levels of cooperation, which we present in detail in the results (e.g., figures 1 and 2).

5. In Data collection, the authors could explain the game procedure more clearly, especially Line 201-204. Does "after all games were played" mean "after all children participated in the game"? During the game, the participant did not know that they will anyway get candies later, regardless of the game results? This thing can be explained more clearly.

This has now been updated (lines 225-229): "The total number of candies earned was given to children after all children participated in the game, which included both the amount each child kept for themselves and resources given to them by other children. To ensure that every child received something, all children were also given additional candies; children playing the games were not aware of these additional candies at the time."

6. In Statistical analysis, it is not so clear which models the authors ran, which models failed due to convergence issues, and which results from which model the authors use for interpretation.

Given the change in analysis to Bayesian models, there were fewer model failures/convergences issues. We hope this is now clearer in the revised manuscript.

7. The subtitles in the Result section: "Amount shared" and "Partner Choice Analyses" can be changed "The level of sharing" and "Sharing partners".

This has been updated.

8. In Result, Table 1 is not intuitive and not easy to understand. I suggest to separate columns, one for Number shared, and another for Number of unique recipients.

Table 1 has been revised accordingly.

9. In Discussion, I suggest the authors to discuss Agta sharing norms among adults, for example regarding generalised reciprocity and kinship, as well as to add some description on food sharing norms in Agta's daily lives.

A section on this has been added to the discussion (lines 554-558): "Regardless of the specific mechanisms by which Agta children acquire these cooperative behaviours, patterns of cooperation among Agta children are comparable to those among Agta adults, with considerable variation in cooperativeness between camps and a strong kin bias in sharing

partners (24,52,53).” Note that we have kept this discussion relatively brief to try and avoid increasing the length of the paper too much.

Line 37: Change “cooperative development” to “development of cooperative behaviours” for clarity.

Done.

Line 84: Change “in that” into “in which”.

No longer applicable, as sentence has already changed during revision.

Line 181: Change “to assess cooperation” into “to assess the level and pattern of cooperation”.

Done.

Line 339: Where did these two additional resources come from?

These are predicted values. That is, an increase in adult cooperation by 70 percentage points predicted that children would share an additional two candies (say, from 1 to 3). This has been updated to (lines 400-402): “For context, given a 70-percentage point increase in camp-level adult cooperation, which is the difference between the most and least cooperative camps, children would be predicted to share approximately two additional resources.”

Line 464-467: I would move to the last paragraph, and here I would give more specific summary of what this finding means.

We would prefer to keep this sentence where it is, as it sets up the next few paragraphs of the discussion which explain this summary in more detail. We hope this is okay.
