# Supplementary material for: Cooperation and partner choice among Agta hunter-gatherer children: An evolutionary developmental perspective
Source: PLoS One. 2023 Apr 26;18(4):e0284360. doi: 10.1371/journal.pone.0284360 (PMC10132543; doi:10.1371/journal.pone.0284360)
Supplement: Attachment — Submitted filename: KidsCoopPaper_ResponseToReviewers_Rev2.pdf [file pone.0284360.s004.pdf]

Cooperation and partner choice among Agta hunter-gatherer children: an evolutionary developmental perspective (PONE-D-22-29549R1) – Editor comments

Additional editor comments:

Here are a handful of more specific issues that I think need addressing in the revision:

We thank the editor for their helpful suggestions, which we have addressed below and in the manuscript. The editor's original comments are in black font, and our responses in blue.

Methods

- How was the 'Sex' variable constructed? Was it using self-report data? If so, it might be important to consider using the term 'gender'. If not, it would be great to state why 'sex' is used.

As this variable was based on genealogical interviews with a parent (usually the mother), this reflects the child's assigned sex at birth, so we would prefer to keep this as 'sex' as we did not ask how the child identifies themselves in terms of gender. This has now been updated in the text: "…sex (child's assigned sex at birth, based on parental genealogical interviews; 0 = female; 1 = male)…" (lines 252-253).

- 250-251: I'm guessing that Wright's coefficient of relatedness is used here? How was it calculated, using the kinship2 package? If so, please note this and cite the package.

Yes, that is correct, we used Wright's coefficient of relatedness calculated using the 'kinship2' package. This information has now been added to the manuscript (lines 254-255).

Discussion

Generally, I think that the discussion of results around children's network size and structure could be toned down a little, and use of terminology slightly more refined. First, I think it would be best to add "personal" in front of "networks". This is a minor change, but has important consequences for readers. The present analyses is essentially capturing a partial sample of a network or individuals' ego networks. Therefore, the present data/analyses cannot directly speak to the global structure of children's networks in the population. Given this, it might be considered quite a stretch to conclude that the present findings show that "the structure of [children's] cooperation networks, changes considerably over childhood" (lines 552-554). The structure of children's networks is not something that is being examined here, and this quite conclusive statement begs questions as to what structural properties of a network are observed to change. It seems that the results might speak more

directly to the demographic composition of children's cooperation partners being different across ages.

Yes, we agree that we are measuring personal networks here rather than global network structures - Thanks for the suggestion and clarification. We have amended the manuscript where appropriate to refer to ‘personal sharing networks’ specifically, rather than ‘cooperative networks’ (or just ‘networks’) more broadly. We have also toned down our language to avoid making broader claims than justified in the discussion; for instance, we have changed “the structure of [children's] cooperation networks, changes considerably over childhood” to “the demographic composition of [children’s] personal sharing networks, changes considerably over childhood” (lines 599-561).

Cooperation and partner choice among Agta hunter-gatherer children: an evolutionary developmental perspective (PONE-D-22-29549R1) – Reviewer 2

Reviewer 2 comments:

I am glad to read the revised manuscript, as the authors improved their statistical analysis methods more rigorously, and it reads well. It is a solid and interesting paper which will contribute to this field.

We thank the reviewer for taking the time to review our paper again, and are delighted that we have addressed most of the reviewer's previous concerns. We hope that our revised manuscript addresses the reviewer's remaining points. The reviewer's original comments are in black font and our responses in blue.

My one remaining concern is the model results of "Sharing Partner". The model results of "Amount Shared" are clearly found in Table 2, but I cannot find the main result tables for "Sharing Partner", also not in the supplementary information either, if I understood correctly. In Lines 466 , 473, 477, 478, authors showed the estimates and 95% confidence intervals, where can the readers find these results in the model tables? I found only Table S5-S9 for additional analyses, but not the main results. In general, compared to the "Amount Shared", description of analysis methods and results of "Sharing Partner" are not clear. Hence, I recommend the authors to add a model result table of sharing partner in the main manuscript.

For these 'Sharing Partners' analyses, all of the key information from these models is reported in the main text (e.g., comparing average relatedness to camp-mates vs nominees on line 471, and associations between age and sex with recipient relatedness on lines 477-483). We do not feel that an additional table repeating this would be necessary, as it would provide no additional information.

And below I have some more minor comments to make the paper clearer.

Line 69: Authors can add reference Jang et al., 2022 on children help mothers increase food returns during foraging.

Jang, H., Janmaat, K. R., Kandza, V., & Boyette, A. H. (2022). Girls in early childhood increase food returns of nursing women during subsistence activities of the BaYaka in the Republic of Congo. Proceedings of the Royal Society B, 289(1987), 20221407.

Now added.

Line 75-76: Could you explain what patterns of cooperation are similar across societies until middle childhood?

We have now expanded this sentence to say "…patterns of costly cooperation were similar in all populations until middle childhood, with levels of cooperation decreasing between the ages of 3 and 6-8 years, after which societies diverged to approximate population-specific adult levels of cooperation" (lines 76-77).

Line 80: Authors can add reference Lew-Levy et al., 2018, a review paper on how hunter-gatherer children learn social norms.

Lew-Levy, S., Lavi, N., Reckin, R., Cristóbal-Azkarate, J., & Ellis-Davies, K. (2018). How do hunter-gatherer children learn social and gender norms? A meta-ethnographic review. Cross-Cultural Research, 52(2), 213-255.

Now added.

Line 83: Add reference (11) in the end of sentence.

Now added.

Line 92: Change "For instance, say that young children may preferentially only share…." to "For instance, young children may preferentially only share….".

Now updated.

Line 115: Change "Partner choice is unlikely to…" to "The pattern of partner choice is unlikely to…".

Now updated.

Line 123: Authors can add references Lew-Levy et al., 2017; 2018, two review papers on how hunter-gatherer children learn social norms and subsistence skills.

Lew-Levy, S., Reckin, R., Lavi, N., Cristóbal-Azkarate, J., & Ellis-Davies, K. (2017). How do hunter-gatherer children learn subsistence skills? A meta-ethnographic review. Human Nature, 28, 367-394.

Lew-Levy, S., Lavi, N., Reckin, R., Cristóbal-Azkarate, J., & Ellis-Davies, K. (2018). How do hunter-gatherer children learn social and gender norms? A meta-ethnographic review. Cross-Cultural Research, 52(2), 213-255.

Now added.

Line 136: This sentence is repetitive with previous paragraphs, the authors can maybe shorten this paragraph and merge it with the previous paragraph, probably by saying: "Therefore, to compare overall levels of cooperation as well as to explore who children share with and whether this pattern changes with age, incorporating partner choice into experimental designs is necessary. While partner choice has been increasingly investigated among adults (23, 36-39), there are only few studies applying such methods with children, especially in small-scale societies.".

This has been updated as suggested.

Line 153: Authors can change "….adult-levels of cooperation (previous research…… (24))" to, "….adult-levels of cooperation, as previous research…… (24)".

Now updated.

Line 178: Can we still call the camp with 26 houses with 156 individuals as camp, but not a village?

We agree that this is a rather large camp(!), but for consistency throughout the paper we would prefer to refer to all Agta settlements as 'camps'.

Line 217: Authors can change "…both of these are decisions made repeatedly by children every day…." to "…both of these are decisions made repeatedly by children in their daily lives…."

Updated as suggested.

Line 224: Authors can probably delete "after which the game was played"? Did authors ask these questions also before the game was played?

These questions were only asked before the games were played (to ensure the children understood the rules of the game), and were not asked afterwards. To make this clearer, we have updated this sentence to: "Before playing the game, children were asked if they understood the rules and if they had any questions. After this, the game was played." (lines 225-227).

Line 233: Authors can change to "too shy and/or too young to fully understand".

Now updated.

Line 257: Authors can change to “the causal relations between variables were considered”.

Now updated.

Line 270: Authors can change to “Our hypothesised causal structure suggest that…..”.

Now updated.

Line 275, 375: Using the terminology “null linear model” or “null multi-level model” can make readers confused with null hypothesis testing, so I would suggest to use “a baseline model which accounts for multilevel structure of data with random effects, but includes no covariates” instead of “null model” throughout the paper.

This is a good point, thanks for raising, and we agree this could potentially be confusing for readers. While the reviewer’s suggestion is accurate, it is rather wordy; to save space, we have instead replaced ‘null multi-level model’ with ‘intercept-only multi-level model’ (lines 278 and 380) – We hope this is okay and avoids any potential confusion.

Line 276: “camp ID” and “household ID” for precision.

Now updated.

Line 301: “The first set of analyses aimed to assess whether….”.

Now updated.

Line 313: Do authors mean “When only one of the parents, either mother or father, participated, the score from the one-parent was used as an average score in the combined parental model.”?

Yes, thanks. This has been updated accordingly.

Line 314: Authors investigated specifically how children shared candies to others, therefore, I would suggest using “sharing networks” instead of “cooperative networks” throughout the paper.

This has now been updated to ‘personal sharing networks’ throughout the paper, based on both the reviewer’s and the editor’s suggestions.

Line 327: How can authors still have “major convergence issues”?

In some of the Sharing Partners Analyses the inclusion of both participant ID and recipient ID as random effects in the same model resulted in major convergence and estimation issues and poor model fit, as indicated by large r-hat values (around 3 or 4, rather than values of ~1 indicating that the chains had converged). As we explain in the text (lines 331-339), this is likely because participant ID and recipient ID random effects are highly collinear. To avoid this issue, while still capturing most of the non-independence in the data, the results in the manuscript are based on including just participant ID as a random effect.

Line 365: To be more accurate, change to “participant’s age, sex and participant’s relatedness to recipient”.

Now updated.

Line 390-396: This paragraph can still be moved to the end of Statistical Analyses part for Amount Shared.

As this paragraph includes the results of statistical analyses, we feel this fits better where it currently is in the Results section, rather than in the Methods.

Table 3: This can be moved to the Supplementary Information.

In Model specification in Table 3, “childCoop” can be changed to “numShared” to increase accuracy.

We have updated the variable name as suggested. However, we would prefer to keep table 3 in the main text as we feel that these model comparisons testing different age-associations are an important and interesting result, and could easily be missed or overlooked if moved to the supplementary material.

Line 525: “Consistent with the result from previous research across six societies (11),”

Now updated.

Line 529: “middle childhood”, instead of “mid-childhood”

Now updated.

Line 553: I would avoid using the term “the structure of their cooperative networks”, especially as the authors clearly wrote that they did not use social network analyses, and I am also not convinced which network structure they found.

This was also noted by the editor, and has now been updated to “the demographic composition of [children’s] personal sharing networks, changes considerably over childhood” (lines 559-561).

Line 608-623: I think this paragraph can be potentially removed as the authors made sure the children understood the games (see Line 224).

While we did indeed ask whether children understood the game prior to proceeding, and there appeared to be no issues in children’s comprehension of the game, we did not run formal comprehension checks to assess this, which could provide additional confidence in our results (or for future studies using similar methodologies). While we do not believe that this invalidates our conclusions, it is a valid limitation which we feel ought to be acknowledged and addressed (as this was a criticism of a previous reviewer of this paper, it may be a concern to other readers as well, so we feel it is worth noting).
